# Abnormal gamma phase-amplitude coupling in the parahippocampal cortex is associated with network hyperexcitability in Alzheimer’s disease

**DOI:** 10.1093/braincomms/fcae121

**Published:** 2024-04-09

**Authors:** Pooja Prabhu, Hirofumi Morise, Kiwamu Kudo, Alexander Beagle, Danielle Mizuiri, Faatimah Syed, Karunakar A Kotegar, Anne Findlay, Bruce L Miller, Joel H Kramer, Katherine P Rankin, Paul A Garcia, Heidi E Kirsch, Keith Vossel, Srikantan S Nagarajan, Kamalini G Ranasinghe

**Affiliations:** Department of Radiology and Biomedical Imaging, University of California San Francisco, San Francisco, CA 94158, USA; Department of Data science and Computer Applications, Manipal Institute of Technology, Manipal 576104, India; Department of Radiology and Biomedical Imaging, University of California San Francisco, San Francisco, CA 94158, USA; Medical Imaging Business Center, Ricoh Company Ltd., Kanazawa 920-0177, Japan; Department of Radiology and Biomedical Imaging, University of California San Francisco, San Francisco, CA 94158, USA; Medical Imaging Business Center, Ricoh Company Ltd., Kanazawa 920-0177, Japan; Memory and Aging Center, Department of Neurology, University of California San Francisco, San Francisco, CA 94158, USA; Department of Radiology and Biomedical Imaging, University of California San Francisco, San Francisco, CA 94158, USA; Memory and Aging Center, Department of Neurology, University of California San Francisco, San Francisco, CA 94158, USA; Department of Data science and Computer Applications, Manipal Institute of Technology, Manipal 576104, India; Department of Radiology and Biomedical Imaging, University of California San Francisco, San Francisco, CA 94158, USA; Memory and Aging Center, Department of Neurology, University of California San Francisco, San Francisco, CA 94158, USA; Memory and Aging Center, Department of Neurology, University of California San Francisco, San Francisco, CA 94158, USA; Memory and Aging Center, Department of Neurology, University of California San Francisco, San Francisco, CA 94158, USA; Epilepsy Center, Department of Neurology, University of California San Francisco, San Francisco, CA 94158, USA; Department of Radiology and Biomedical Imaging, University of California San Francisco, San Francisco, CA 94158, USA; Epilepsy Center, Department of Neurology, University of California San Francisco, San Francisco, CA 94158, USA; Memory and Aging Center, Department of Neurology, University of California San Francisco, San Francisco, CA 94158, USA; Mary S. Easton Center for Alzheimer’s Research and Care, Department of Neurology, David Geffen School of Medicine, University of California Los Angeles, Los Angeles, CA 90095, USA; Department of Radiology and Biomedical Imaging, University of California San Francisco, San Francisco, CA 94158, USA; Memory and Aging Center, Department of Neurology, University of California San Francisco, San Francisco, CA 94158, USA

**Keywords:** network hyperexcitability, gamma oscillations, magnetoencephalography, phase-amplitude coupling, Alzheimer’s disease

## Abstract

While animal models of Alzheimer’s disease (AD) have shown altered gamma oscillations (∼40 Hz) in local neural circuits, the low signal-to-noise ratio of gamma in the resting human brain precludes its quantification via conventional spectral estimates. Phase-amplitude coupling (PAC) indicating the dynamic integration between the gamma amplitude and the phase of low-frequency (4–12 Hz) oscillations is a useful alternative to capture local gamma activity. In addition, PAC is also an index of neuronal excitability as the phase of low-frequency oscillations that modulate gamma amplitude, effectively regulates the excitability of local neuronal firing. In this study, we sought to examine the local neuronal activity and excitability using gamma PAC, within brain regions vulnerable to early AD pathophysiology—entorhinal cortex and parahippocampus, in a clinical population of patients with AD and age-matched controls. Our clinical cohorts consisted of a well-characterized cohort of AD patients (*n* = 50; age, 60 ± 8 years) with positive AD biomarkers, and age-matched, cognitively unimpaired controls (*n* = 35; age, 63 ± 5.8 years). We identified the presence or the absence of epileptiform activity in AD patients (AD patients with epileptiform activity, AD-EPI+, *n* = 20; AD patients without epileptiform activity, AD-EPI−, *n* = 30) using long-term electroencephalography (LTM-EEG) and 1-hour long magnetoencephalography (MEG) with simultaneous EEG. Using the source reconstructed MEG data, we computed gamma PAC as the coupling between amplitude of the gamma frequency (30–40 Hz) with phase of the theta (4–8 Hz) and alpha (8–12 Hz) frequency oscillations, within entorhinal and parahippocampal cortices. We found that patients with AD have reduced gamma PAC in the left parahippocampal cortex, compared to age-matched controls. Furthermore, AD-EPI+ patients showed greater reductions in gamma PAC than AD-EPI− in bilateral parahippocampal cortices. In contrast, entorhinal cortices did not show gamma PAC abnormalities in patients with AD. Our findings demonstrate the spatial patterns of altered gamma oscillations indicating possible region-specific manifestations of network hyperexcitability within medial temporal lobe regions vulnerable to AD pathophysiology. Greater deficits in AD-EPI+ suggests that reduced gamma PAC is a sensitive index of network hyperexcitability in AD patients. Collectively, the current results emphasize the importance of investigating the role of neural circuit hyperexcitability in early AD pathophysiology and explore its potential as a modifiable contributor to AD pathobiology.

## Teaser

Prabhu *et al*. examined phase-amplitude coupling between gamma amplitude and phase of lower frequencies in patients with Alzheimer’s disease (AD) and their associations with network hyperexcitability. Theta-gamma coupling in AD was reduced in medial temporal regions (parahippocampus), which is the earliest affected region in AD, and was associated network hyperexcitability.

## Graphical Abstract

**Graphical abstract:**
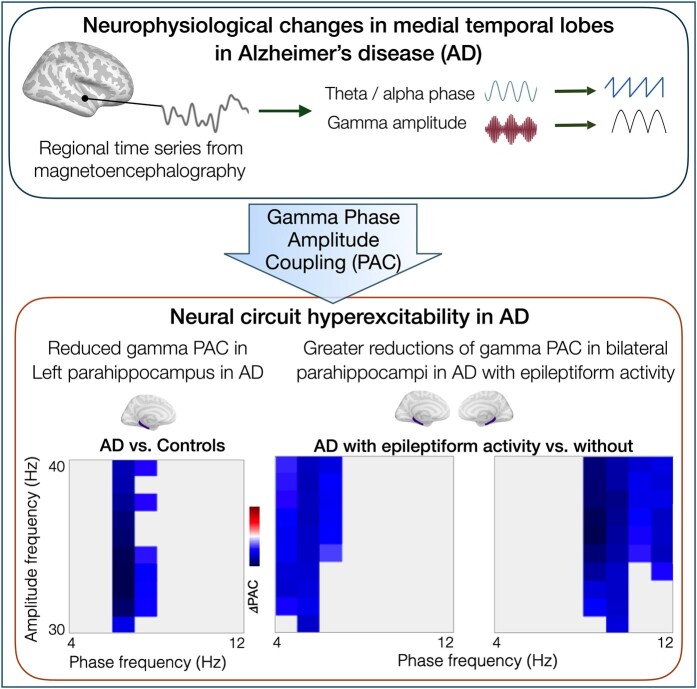

## Introduction

Animal models of Alzheimer’s disease (AD) have demonstrated that network hyperexcitability is strongly associated with AD proteinopathy and behavioural deficits.^1,2^ These studies have identified aberrant neuronal firing as an early phenomenon in AD pathogenesis.^3^ Consistent with these basic science evidence of network hyperexcitability being closely associated with disease pathophysiology, clinical studies in patients have also demonstrated high incidence of epileptic manifestations, either as overt seizures or as subclinical epileptiform discharges in patients with AD.^4-6^ Importantly, neural circuit hyperexcitability is a key mechanism that contributes to AD pathophysiology within regions that are vulnerable to AD-tauopathy.^7^ For example, a transgenic mouse model which overexpressed both human amyloid precursors protein (hAPP) and entorhinal tau showed that amyloid-beta (Aβ) significantly increased the excitability of entorhinal neurons which then facilitated the spread of tau into the downstream hippocampus while attenuation of the hyperexcitability reduced these pathologies.^8^ Patterns of altered local excitability in the medial temporal lobes in patients with AD remains largely unknown.

Neural oscillations represent the composite activity of excitatory firing of principal cells and rhythmic inhibition of interneurons arranged into hierarchical cellular networks. Local excitability of these networks is thought to be best represented in the gamma range frequency (>30 Hz).^9^ Gamma abnormalities are indeed the most frequently reported oscillatory abnormalities in AD transgenic mice.^10,11^ However, in the human brain, a major obstacle in examining gamma band activity from non-invasive neuroimaging is its low signal-to-noise ratio due to the distance of sensors from the cortex and contamination from other muscular activity artefacts.^12^ While increased gamma power is observed in a region-dependent manner when engaged in a cognitive task,^13,14^ during rest, it reaches to very low amplitudes, almost undetectable by standard power density assays. In this context, an effective means to capture gamma activity, even at rest, is cross-frequency coupling between gamma oscillations and lower frequency theta and alpha oscillations.^15,16^ Phase-amplitude coupling (PAC), which specifies the statistical dependence between the amplitude of a high-frequency oscillation (i.e. gamma),^17,18^ with the phase of a low-frequency (i.e. theta or alpha) oscillation is of particular interest here because by modulating the amplitude of the high frequency, the phase of the low-frequency oscillations essentially regulates the excitability of local neuronal firing.^19,20^ Quantification of gamma band cross-frequency-coupling, therefore, not only provides a measure of local neuronal activity but also an index of neuronal excitability within an ensemble regional network. Importantly, PAC may be particularly suited as a robust index of local circuit abnormalities associated with AD pathobiology, which are mostly subclinical manifestations of hyperexcitability where the conventional short duration electrophysiological recordings are less sensitive.

In this study, we sought to examine the local neuronal hyperexcitability using gamma PAC within brain regions vulnerable to early AD pathophysiology—entorhinal cortex and parahippocampus. Specifically, we examined the coupling between amplitude of the gamma frequency (30–40 Hz) with phase of the theta (4–8 Hz) and alpha (8–12 Hz) frequency oscillations, in a well-characterized cohort of AD patients and age-matched controls (AD versus control). We then categorically identified AD patients with and without epileptiform activity—the cardinal clinical manifestations of network hyperexcitability, and examined the theta-gamma and alpha-gamma PAC in patients with and without subclinical epileptiform activity (AD-EPI+ versus AD-EPI−). We hypothesized that abnormal gamma PAC would indicate relative patterns of network hyperexcitability within the medial temporal lobes of entorhinal and parahippocampal cortices associated with AD pathophysiology.

## Materials and methods

### Participants

We used a well-characterized cohort of AD patients that was described in a previous investigation^21^ (*n* = 50 AD patients; age, 60 ± 8 years; Supplementary tables 1 and 2) and 35 age-matched controls (age, 63 ± 5.8 years). All patients met National Institute of Ageing-Alzheimer’s Association criteria with positive biomarkers, including cerebrospinal fluid (CSF) and/or positive amyloid reading in positron emission tomography (PET), or histopathological confirmation at autopsy (Supplementary table 3).^22,23^ In long-term monitoring by video EEG (LTM-EEG) and magnetoencephalography (MEG) evaluations, 20 AD patients had subclinical epileptiform activity (AD-EPI+), and the remaining 30 did not show epileptiform activity (AD-EPI−). Age-matched controls were recruited based on the criteria: normal cognitive performance; normal magnetic resonance imaging (MRI); and the absence of neurological, psychiatric or other major illnesses. All the participants were recruited through the University of California San Francisco (UCSF) Memory and Aging Center. Informed consent was obtained from each participant or their surrogate decision makers. The study was approved by the UCSF Institutional Review Board.

### Data acquisition

Each patient underwent overnight LTM-EEG, which was recorded using silver cup electrodes placed in 10–20 electrode array, with 3 minutes of hyperventilation. LTM-EEG was acquired at the Clinical and Translational Science Institute Clinical Research Center at Moffitt Hospital at UCSF. On the following day morning (between 9.30 and 11.00 AM), each AD patient underwent 1 hour resting-state MEG recording and simultaneous 21-lead EEG recording. Patients were instructed to lie supine with eyes closed during data acquisition and the 60 second data epoch for quantitative analysis was selected during the awake, eyes-closed, resting period of data collection. Whole-head CTF MEG system with 275 axial gradiometers was used (sampling rate of 600 Hz). To co-register MEG with brain MRI and to obtain a head position with respect to the sensor array, fiducial coils were placed at nasion, left pre-auricular and right pre-auricular points. Epileptiform discharges were identified using the LTM-EEG and M/EEG in AD patients as described previously.^4,21^ Participants were in a supine position with their eyes closed during the MEG acquisition. Out of 35 controls, 17 were monitored based on LTM-EEG and M/EEG; 8 were monitored based on 1-hour resting-state M/EEG; and 10 were monitored with 10-minutes of resting-state MEG. None of the control participants showed evidence of epileptiform manifestations within their respective electrophysiological recordings. All participants structural MRI were acquired and then used to generate individualized head models for MEG source reconstruction.

### Source reconstruction of MEG data

**Figure 1:**
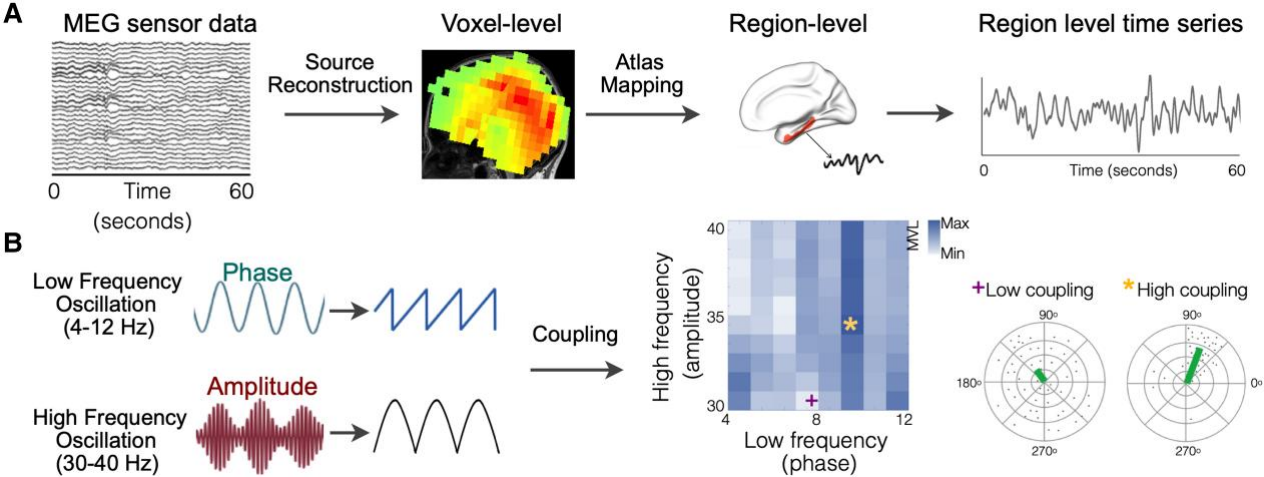
**Estimation of gamma band PAC**. MEG source-reconstructed region-level time series is mapped onto atlas parcellations to obtain time series for each cortical region of L and R entorhinal and L and R parahippocampal cortex (**A**). Each region-level time series was filtered at 4–12 Hz and 30–40 Hz to get phase and amplitude signals, respectively. Then, the coupling strength is measured using the mean vector length between the phase of 4–12 Hz signal and the amplitude of 30–40 Hz signal (**B**). Idealized depiction of high coupling and low coupling is shown in colour gradient on the PAC grid. Polar plots correspond to data points of complex composite signals for the low and high PAC identified on the grid. Mean of these points (mean vector length) is represented in the green line. High coupling is represented by a large mean vector length and low coupling is represented by a small mean vector length. (PAC, phase-amplitude coupling).

We selected a contiguous 60-second data epoch from the initial segment of the awake-resting MEG recording. The awake-resting state of each participant was further assured by inspecting the power spectral density of the 60-second dataset. Artefact detection was based on visual inspection of channels and trials, ensuring that the peak magnetic field of the data did not cross 1pT. Data that were beyond this threshold was subjected to established denoizing algorithms.^24^ Spatiotemporal estimates of neural sources were generated using time-frequency analyses implemented in the Neurodynamic Utility Toolbox for MEG (NUTMEG; http://nutmeg.berkeley.edu)^25^. Source-space reconstructions of MEG sensor data were generated using a Linear-Constrained Minimum Variance (LCMV) beamformer with a 10 mm lead field. Voxel-based source signals were mapped to the Desikan-Killany (DK) atlas^26^ to obtain regional time series (Fig. 1A). To test our main hypothesis, we used *apriori* selected, known medial temporal lobe regions that show highest vulnerability to early AD pathophysiology. In the DK atlas parcellations these regions included the right (R) and left (L) entorhinal cortices, and R and L parahippocampal cortices.

### Phase-amplitude coupling

We estimated PAC using coupling strength (mean vector length) between the phase of lower frequency (LF) oscillation (4–12 Hz) and amplitude of high-frequency (HF) oscillation (30–40 Hz) for every 1 Hz frequency bin (Fig. 1B). The low-frequency range of 4–12 Hz is of particular relevance to AD pathophysiology as these include the frequency bands that show signature spectral changes in AD. Specifically, it is well described that in patients with AD the 4–8 Hz range oscillatory activity, which captures the theta range activity is increased while the 8–12 Hz alpha oscillatory activity is reduced, compared to healthy elderly individuals.^27^ Previous studies have further demonstrated that these frequency-specific oscillatory changes are closely associated with amyloid and tau accumulations in AD.^28-31^ Apart from its relevance to AD, previous physiological investigations which established PAC as a cross-frequency coupling metric has demonstrated that gamma PAC is robustly detected with the amplitude of theta and alpha range frequencies.^18,32^ For this analysis, we first generated a grid of 80 PAC combinations of phase and amplitude pairs. For each of four region’s time series, we computed the modulation index^18^ between the phase of theta (4–8 Hz) or alpha (8–12 Hz) and the amplitude of gamma (30–40 Hz). The modulation index was computed as the complex-valued composite signal, which is a combination of amplitude time series (30–40 Hz) with phase time series (4–12 Hz). The amplitude time series (A_H_(t)) was obtained by extracting the envelope (or amplitude) by applying Hilbert transform to the high-frequency bandpass signal (30–40 Hz). The phase time series ($\Phi_{L}(t)$) was obtained by extracting the phase by applying Hilbert transform to the low-frequency bandpass signal (4–12 Hz). The complex-valued composite signal was estimated as Z(t) = A_H_(t) $e^{i\Phi_{L}(t)}$. The coupling between the A_H_ and Ф_L_ is the degree of asymmetry of the probability density function of Z(t) which was obtained by measuring the mean of Z(t) (denoted by M_raw_). The mean of Z(t) was first normalized to examine the joint distribution of A_H_ and Ф_L_ by considering the forms of the marginal distribution of A_H_ alone and Ф_L_ alone. To accomplish this, we compared the mean with the set of means from surrogates (*n* = 200). Surrogates were generated by introducing time lag τ such that Z(t, τ) = A_H_(t + τ) $e^{i\Phi_{L}(t)}$. For large τ, the asymmetry in Z(t, τ) in distribution was due to Ф_L_ and A_H_ alone determined how far points fall from the origin. The normalized (or *z*-scored) mean was M_norm_ = (|M_raw_|-μ)/σ, where μ and σ is the mean and standard deviation of the surrogate’s length, respectively. The normalized phase angle, which defines the temporal relationship between the low-frequency and the gamma signal (Ф_norm_) was computed from the complex-value M_raw_. Thus, the coupling strength between the time series of two frequency bands was indicated as M_norm_ and the phase angle between them was indicated as Ф_norm_. Idealized depiction of high coupling and low coupling is shown in Fig. 1B. We generated polar plots where each point corresponds to complex values of the composite signal (Fig. 1B). To compute PAC from the regional time series, we used the EEGlab event-related PAC toolbox (https://github.com/sccn/PACTools).

### Statistical analysis

We examined the group differences between AD and age-matched controls, and AD-EPI+ and AD-EPI−. To correct for false positives arising from multiple comparisons in the phase-amplitude grid (80 elements) per region, we used a permutation-based cluster test. Independent *t*-tests were calculated for every PAC value on the grid at the region-level between groups. *T*-values that were adjacent in both phase and amplitude frequency below a cluster alpha threshold of 5% were added up to create clusters. After 1024 permutations (or repetitions) of randomly alternating PAC values within each region, a permutation distribution was calculated. By contrasting the cluster statistic with the random permutation distribution, the permutation *P*-value was found. When the total of the *t*-values was more than 95% (or *P* < 0.05) of the permuted distribution, the observed clusters were regarded as statistically significant. We also calculated the mean phase angle as the average across the PAC-grid combinations that was statistically significant in mean vector length between group contrasts (i.e. AD versus age-matched controls and AD-EPI− versus AD-EPI+), for each subject. The mean phase angle was obtained by averaging the complex values and then calculating the angle of the resultant complex value. We also compared the phase angle of the PAC-grid combinations that showed group difference in mean vector length, using unpaired *t*-tests.

## Results

### Participant demographics

Clinical and demographic characteristics of AD patients and controls used in the current study have been detailed in a previous report^21^ and also provided in the Supplementary table 1. AD patients with and without epileptiform activity were matched in their age and other demographics including age, sex and education (Supplementary table 1; AD-EPI+: age, 59.9 ± 6.7 years, female sex, 12 (60%), education, 17 ± 2.7 years, and AD-EPI−: age, 60.7 ± 8.3 years, female sex, 17 (56.7%), education, 15.7 ± 2.6 years), as well as in clinical and cognitive characteristics (Supplementary table 2).

### Reduced theta-gamma coupling in the left parahippocampal cortex in AD

**Figure 2:**
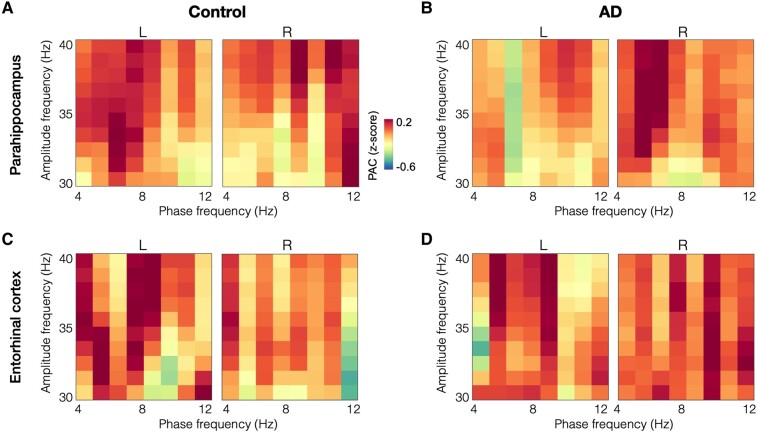
**Gamma band PAC in patients with AD and age-matched controls in parahippocampal and entorhinal cortices**. Gamma PAC within parahippocampal cortices, especially the left parahippocampus showed reductions in patients with AD than controls (**A-B**). Gamma PAC within entorhinal cortices showed similar patterns in age-matched controls and in patients with AD (**C-D**). (AD, Alzheimer’s disease; PAC, phase-amplitude coupling).

**Figure 3:**
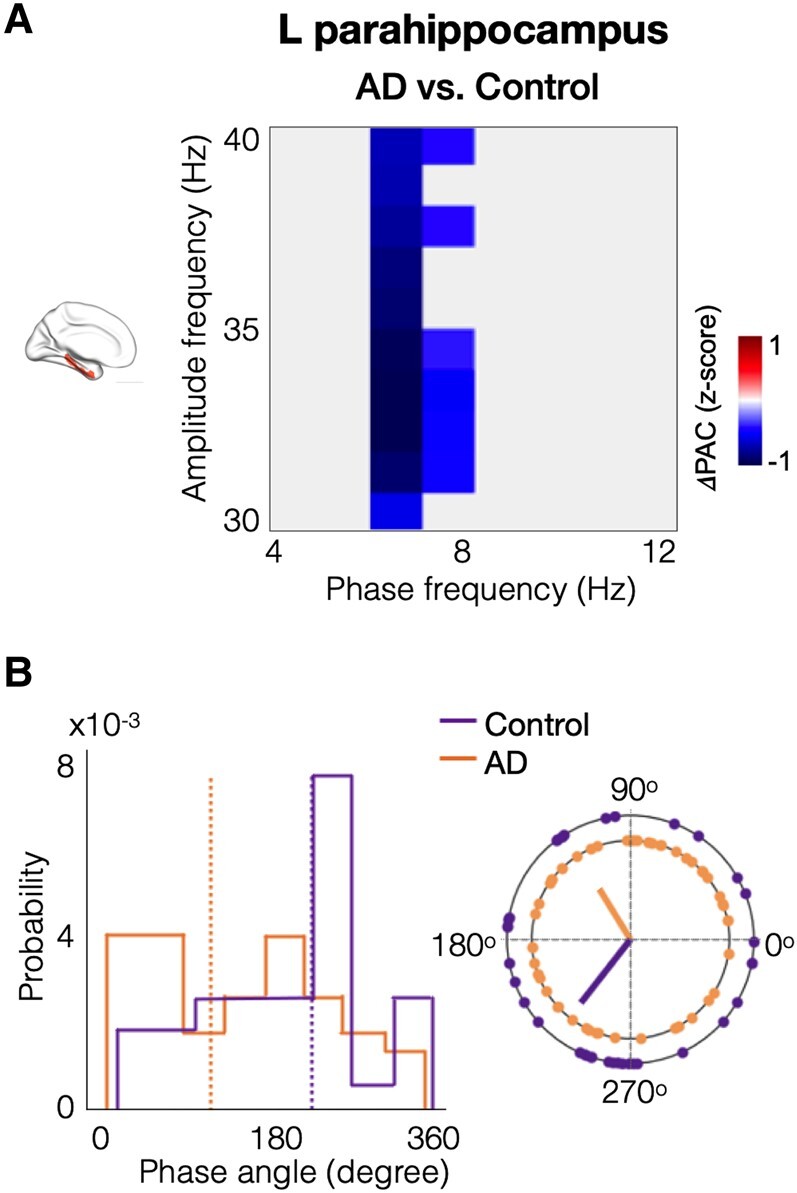
**Patients with AD showed reduced gamma PAC within left parahippocampus**. Patients with AD showed reduced coupling of gamma amplitude and theta (6–8 Hz) phase in the left parahippocampal cortex compared to age-matched controls (**A**). The blue-coloured phase-amplitude coupling range showed statistically significant reductions in AD patients versus age-matched controls in the left parahippocampus (*t* = 6.827, *P* = 0.037). The phase angle distribution in the left parahippocampal region, within the significant phase-amplitude coupling range (as indicated in subplot **A**), showed that AD patients (orange dotted line; mean phase angle = 121.7°) are leading age-matched controls (purple dotted line; mean phase angle = 231.8°) (**B**). (AD, Alzheimer’s disease; PAC, phase-amplitude coupling).

The patterns of gamma PAC within the parahippocampal cortices showed reductions in patients with AD, especially with in the left parahippocampus, compared to age-matched controls (Fig. 2A-B), while the entorhinal gamma PAC seems similar across both groups (Fig. 2C-D). Specifically, in the left parahippocampal cortex, patients with AD showed reduced theta-gamma PAC compared to age-matched controls, where gamma amplitude showed reduced coupling within the 6–8 Hz oscillatory range (Fig. 3A-B). The average normalized PAC of gamma amplitudes that showed reduced coupling for the 6–8 Hz phase at cohort level was −0.235 for AD patients and 0.312 for controls (Fig. 3A, *P* = 0.037). The mean phase angle was computed as the average across the PAC-grid combinations that showed a group difference between AD and controls, for each subject’s left parahippocampal region. The mean phase angle (dotted line in Fig. 3B) in patients with AD was 122° preceding the phase angle in age-matched controls at 232° (Fig. 3B). Although not significantly different from each other, both groups were closer to 90° or 270° than 0° or 180° phase (*t* = 0.3984, *P* = 0.6914). In summary, the coupling strength in the left parahippocampus is significantly reduced while the timing of neuronal activity patterns that contribute to PAC are not significantly altered in AD.

### Reduced gamma PAC in bilateral parahippocampal cortices in AD-EPI+

**Figure 4:**
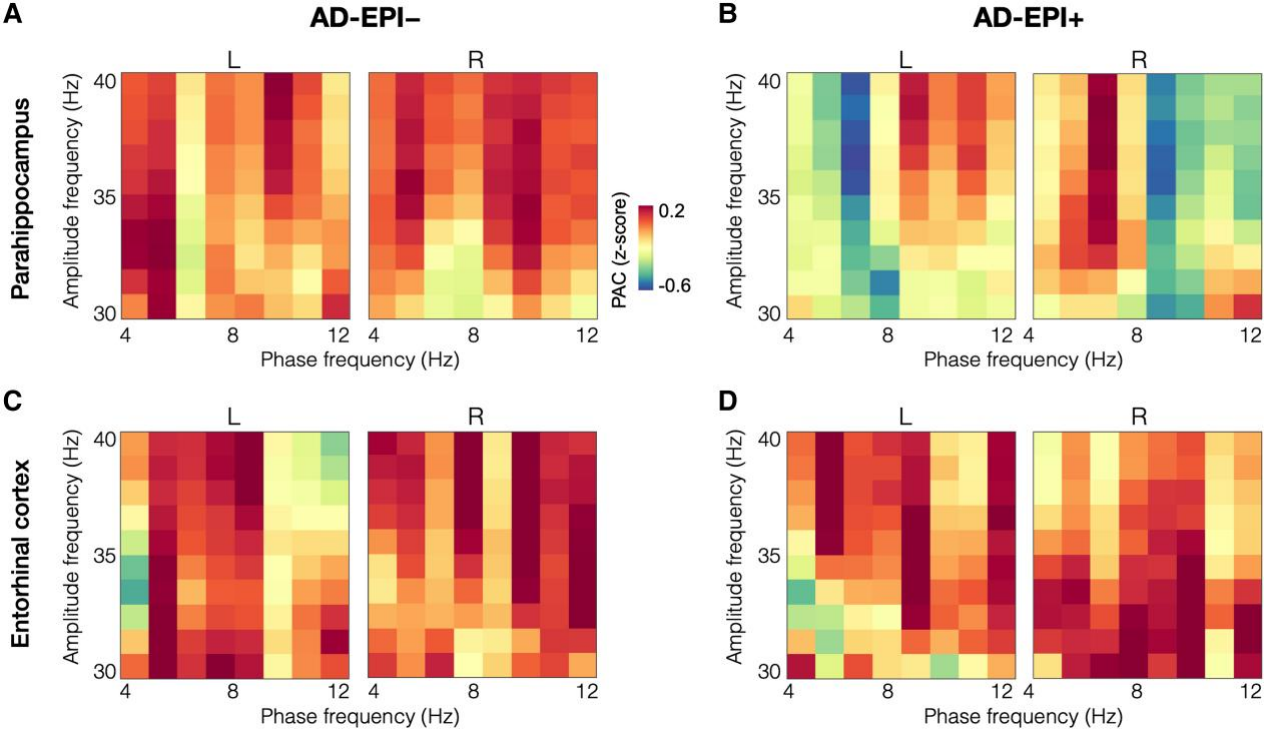
**Gamma band PAC in AD-EPI− and AD-EPI+ in parahippocampal and entorhinal cortices**. Gamma PAC within both left and right parahippocampal cortices, showed reductions in patients with AD-EPI+ compared to patients with AD-EPI− **(A-B)**. Gamma PAC within entorhinal cortices showed similar patterns in AD-EPI− and AD-EPI+ **(C-D)**. (AD, Alzheimer’s disease; PAC, phase-amplitude coupling).

**Figure 5:**
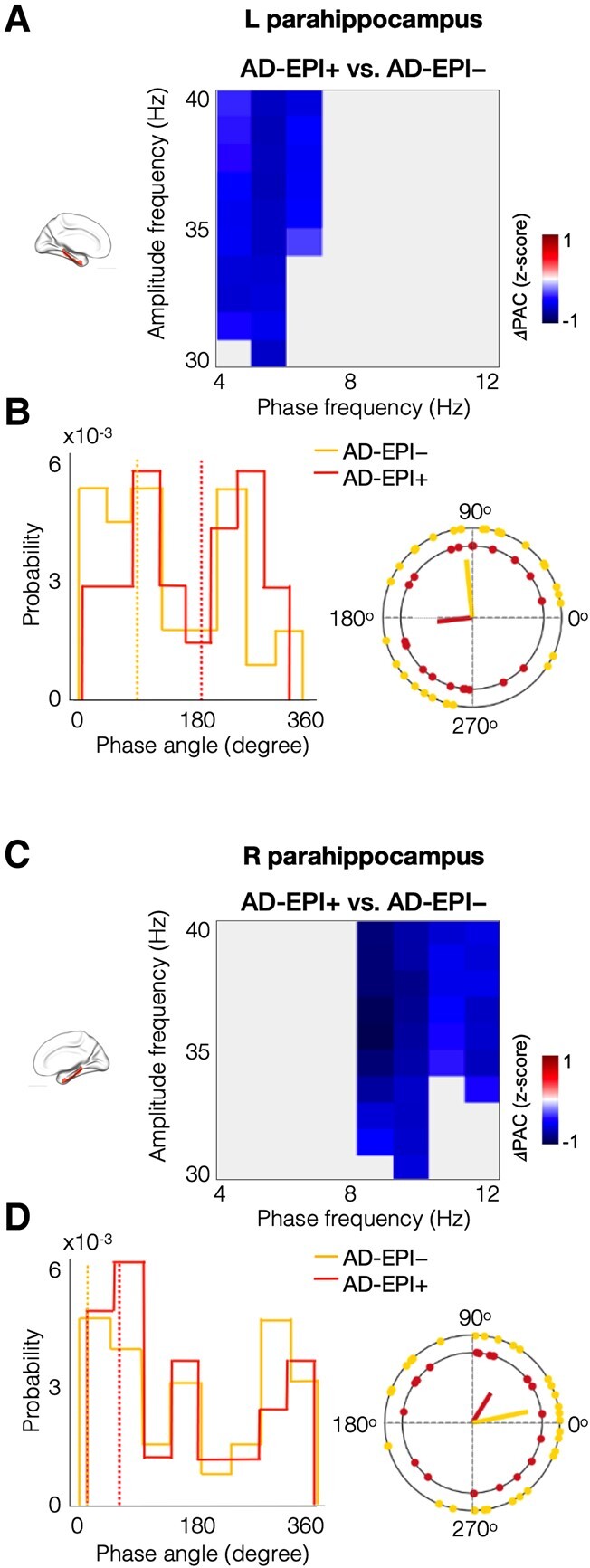
**Patients with AD-EPI+ showed reduced gamma PAC in bilateral parahippocampal cortices compared to AD-EPI−**. Patients with AD-EPI+ showed reduced theta-gamma PAC in the left parahippocampal region (**A**). The blue-coloured phase-amplitude coupling range showed statistically significant reductions in AD-EPI+ patients versus AD-EPI− patients in the left hippocampus (*t* = 4.286, *P* = 0.039). The phase angle distribution in the left parahippocampal region, illustrated in subplot **B**, within the significant phase-amplitude coupling range (indicated in blue in subplot **A**), showed that AD-EPI− (yellow dotted line; mean phase angle = 95.8°) patients are leading AD-EPI+ patients (red dotted line; mean phase angle = 186.7°). Patients with AD-EPI+ showed reduced alpha-gamma PAC in the right parahippocampal cortex (**C**; *t* = 5.295, *P* = 0.008). The phase angle distribution in the right parahippocampal region, within the statistically significant phase-amplitude coupling range (as indicated in blue in subplot **C**), showed that AD-EPI− (yellow dotted line; mean phase angle = 11.5°) are leading AD-EPI+ patients (red dotted line; mean phase angle = 57.8°; **D**). (AD, Alzheimer’s disease; AD-EPI+, AD patients with epileptiform activity; AD-EPI−, AD patients without epileptiform activity; PAC, phase-amplitude coupling).

Parahippocampal cortices showed greater reductions in AD-EPI+ compared to AD-EPI−, while entorhinal cortices in both groups showed similar patterns (Fig. 4). Specifically, AD-EPI + showed reduced gamma PAC in the left parahippocampus (Fig. 5A-B, *P* = 0.039) as well as in the right parahippocampus (Fig.5 C-D, *P* = 0.008), compared to AD-EPI−. In particular, within the left parahippocampus, the gamma amplitudes showed reduced coupling with the phase of 4–7 Hz range oscillations indicating reduced theta-gamma PAC (Fig. 5A-B), whereas in the right parahippocampus the gamma amplitude showed reduced coupling with the phase of 8–12 Hz range of oscillations indicating reduced alpha-gamma PAC (Fig. 5C-D). Average phase angles were computed across the range of PAC-grid combinations that showed significant group differences between EPI+ and EPI− patients within each respective anatomical regions (i.e. theta-gamma PAC grid combinations in left parahippocampus; alpha-gamma PAC grid combinations in right parahippocampus). The mean phase angle (yellow dotted line in Fig. 5B and Fig. 5D) in AD-EPI− was at 96° and at 11° in the left and right parahippocampus, respectively. The mean phase angle of AD-EPI+ (red dotted line in Fig. 5B and Fig. 5D) although lagged the phase angle of AD-EPI− in both left and right parahippocampi showing 187° and 58°, respectively, these differences were not statistically significant (Left parahippocampus: *t* = −0.6742, *P* = 0.5034; Right parahippocampus: *t* = 0.5402, *P* = 0.5916).

## Discussion

In this study, we examined the local neuronal activity within gamma oscillations by quantifying the coupling between gamma amplitude and the phase of theta and alpha oscillations, in a well-characterized cohort of AD patients and age-matched controls. We found that patients with AD have reduced gamma coupling compared to controls in the left parahippocampus while AD-EPI+ patients showed greater reductions in gamma coupling within bilateral parahippocampal cortices compared to AD-EPI−. In contrast to parahippocampal cortices, entorhinal cortex did not show altered gamma oscillations either in AD versus controls or in AD-EPI+ versus AD-EPI−. Collectively our findings demonstrate: (i) region-specific alterations in gamma PAC suggesting regional vulnerabilities of network hyperexcitability within the medial temporal lobes in AD and (ii) greater gamma PAC deficits in AD-EPI+ suggesting that gamma PAC is a sensitive index of network hyperexcitability in AD.

### Hyperexcitability within medial temporal lobe subregions in AD

The current study demonstrated altered gamma PAC in patients with AD within parahippocampus but not within entorhinal cortex, compared to age-matched controls. This regional selectivity within medial temporal subregions is particularly intriguing given the regional specificity of age-related tauopathy versus AD-related tauopathy.^33,34^ The emergence of tau-PET has demonstrated the patterns of progressive tau accumulation in healthy aging and in individuals who have Aβ accumulation in the brain. These studies show that while aging-related accumulation of tau, also known as primary age-related tauopathy, is mostly restricted to the entorhinal cortex, spread of tau beyond entorhinal cortex and into the adjacent parahippocampal regions only happens in the presence of Aβ indicating AD neuropathological changes.^35-37^ In this context, altered hyperexcitability in AD patients in the parahippocampus but not in the entorhinal cortex compared to healthy aging pose the question whether neuronal hyperexcitability play a role in the progression of AD-tauopathy beyond entorhinal regions. Indeed, animal models have shown compelling evidence that locally increased neuronal activity stimulates release of tau and enhances tau pathology.^7,8,38^ The current study also demonstrated that patients with AD-EPI+ have greater reductions of gamma PAC involving bilateral parahippocampal cortices, suggesting a possible contributory role of hyperexcitability to the faster rate and more severe cognitive loss associated with AD-EPI+ phenotype.^4,39^ While the relationships between regional patterns of medial temporal lobe hyperexcitability and AD-tauopathy is yet to be demonstrated in human neuroimaging studies, the current results emphasize the importance of investigating the role of neural circuit hyperexcitability in early AD pathophysiology and explore its potential as a modifiable contributor.

### PAC and hyperexcitability in AD

Investigations in AD transgenic mice demonstrate that increased seizure susceptibility and network hyperexcitability are associated with early amyloid and tau accumulations.^10,40^ Consistent with such evidence, we have previously demonstrated an increased incidence of subclinical epileptiform activity in patients with AD^4^ and that AD-EPI+ patients have greater reductions in alpha and greater increases in delta-theta neuronal synchrony than age-matched controls.^21^ The mechanistic relationships between these low-frequency oscillatory abnormalities and network hyperexcitability, however, still remains incompletely understood. In contrast, many basic science epilepsy models and AD models have explored the relationship between the coupling of low-frequency phase to gamma amplitude and epileptogenic phenomenon in local neural circuits. The current results showing reduced gamma coupling in patients with AD and the greater degree of such deficits in AD-EPI+ brings a step closer in understanding the mechanistic relationship between network hyperexcitability and neural oscillatory changes. A rodent model of temporal lobe epilepsy (TLE) demonstrated reduced theta-gamma coupling in the dorsal hippocampus in TLE rats during the interictal period and that impaired inhibitory activity of parvalbumin (PV) basket cells as the major contributory cause for this rhythmopathy.^41^ A mouse model with focal knock-down of SCN1A gene, which encode the Nav1.1 voltage-gated sodium channel in the dorsal hippocampus demonstrated reduced theta-gamma coupling in hippocampus.^42^ In particular, the Nav1.1 knock-down mice showed abnormal spatial memory performance and affected both pyramidal neuronal firing as well as fast-spiking inhibitory interneurons. Indeed abnormal Nav1.1 activity in PV basket cells have been identified as one of the possible contributory causes to network hyperexcitability in transgenic AD mice.^11^ While much of the details of cellular and molecular details of these relationships are yet to be identified, the current results clearly emphasize that abnormal gamma PAC is a key manifestations of altered neural syntax closely coupled to epileptogenic processes associated with AD pathophysiology. Diminished gamma coupling physiologically may have clinical influences on memory and other cognitive functions in AD populations.

Compared to the strength of coupling in basic science disease models, much less is known about the phase angle of coupling in PAC. Under physiological conditions, however, compelling evidence support distinct roles of neuronal activity that occur during the peak and the trough of low-frequency phase modulations such as theta. For example, in awake active rodents hippocampal place cell firing is actively coupled to the peak of the theta phase.^43^ It has been shown that maximum depolarization of place cells occur at the peak of the theta cycle which facilitate long-term-potentiation, whereas firing in the theta trough, where depolarization is not optimized induce long-term-depression, in hippocampal circuits.^44-46^ Although, theta-gamma coupling in resting-state brain may have its own physiological significance from the behaviourally active brain, the phase angle of coupling is still likely to play an important role. Although the phase angles were not significantly different between patients with AD and controls in the selected regional analysis of gamma PAC in our investigation, we believe this is an important area that warrants further investigation in AD. It is likely that the altered timing of neural activity may be associated with AD pathobiology in general, although not necessarily a metric that is indexing hyperexcitability in AD.

### Gamma oscillatory changes in AD

Abnormal spectral signatures of increased delta-theta oscillatory activity and reduced alpha and beta oscillatory activity are well documented in clinical AD populations.^27,28^ Abnormalities in the gamma band oscillatory activity, in contrast, have been difficult to capture, owing to its low signal-to-noise ratio in the spectral power density assay of the resting human brain. In transgenic AD mice, gamma oscillatory activity is consistently reduced^11,47^ including reduced PAC of theta phase and slow gamma power.^48,49^ Findings from human imaging studies show variable results. In one study, widespread reductions in gamma synchronization in patients with AD were reported from whole brain MEG sensor-space analysis,^50^ while the region of interest-based evaluation of frontal/prefrontal cortices using combined transcranial magnetic stimulation and EEG found reduced gamma power in patients with AD.^51^ On the other hand, high midline gamma coherence in EEG was associated with a greater incidence of conversion from mild cognitive impairment (MCI) to AD,^52^ while greater 40 Hz steady-state activity in EEG was found in AD compared to MCI.^53^ Apart from methodological differences, these latter studies could have been influenced by limited spatial resolution and poor source localization ability of EEG local field potential signals. Our findings showing reduced theta/alpha-gamma coupling delivered through high spatial resolution MEG source localization techniques are consistent with previous MEG findings^50,54^ as well as the collective findings from AD transgenic mice studies reporting reduced gamma oscillatory activity in AD. A recent MEG clinical investigation on patients diagnosed with MCI also showed that gamma band functional connectivity in the right temporal cortex was significantly reduced in those who were positive for epileptiform activity than those without.^55^ It is likely that gamma oscillatory activity, which represent the collective firing of local neuronal ensembles are sensitive to the spatial resolution of the imaging modality. Nonetheless, both basic and clinical science investigations provide the collective insight that activity within local circuits are significantly altered in AD, while the current study extend these insights indicating their potential mechanistic links to network hyperexcitability in AD.

The current study demonstrated reduced gamma PAC in AD in the left parahippocampus, but not in the right parahippocampus. This finding may likely be contributed by the fact that the current cohort consisted ∼90% of early-onset AD patients and ∼10% atypical AD presentations. Previous studies have shown a left hemispheric predominance over the right hemisphere in neuronal loss as well as in tau accumulation, in AD patients whose clinical disease onset was before 65 years of age.^56,57^ Among atypical AD, the patients presenting with the clinical syndrome of logopenic variant of primary progressive aphasia show a selective bias for the involvement of the left hemisphere.^58^ While increased vulnerability of the left hemisphere is consistent with the left lateralized gamma PAC reductions in the current study, regional associations of local neural circuit abnormalities and AD pathophysiology warrant further investigation.

### PAC as a measure of local network dysfunction

Synchronized neural oscillations have been proposed as an effective mechanism of network communication in the brain.^59^ While low-frequency oscillations, like theta, are specially adapted for long distance synchronizations, faster oscillations, such as gamma, represent ensemble neuronal activity over comparatively small spatial scales.^15^ When moving from a local to a global scale, interactions between low- and high-frequency oscillations provide a mechanism to integrate these neural processes along the spatial hierarchy of brain activity.^15^ When gamma oscillations appear during a specific phase cycle of the low-frequency oscillation, attracting groups of cells actively engaged at that moment, a synchronized low-frequency oscillation will help to combine these spatially distributed, locally functional activity patterns. It has also been suggested that different low-frequency phases coupled to gamma oscillations may encode distinct information.^60^ In this scheme, the cycle of lower oscillation can be divided into time slots and the coupled gamma oscillatory activity within each epoch encodes distinct representations of local neuronal activity.^61,62^ PAC, which links the phase of the low-frequency neural oscillation with the amplitude of the faster neuronal oscillation has been shown to be a highly reliable index and not only facilitates the separate spatially distributed cortical networks operating in parallel,^17^ but also provides the explanation of ‘neuronal communication through neuronal coherence’.^63^ PAC, therefore, is an attractive metric to quantify a unique aspect of network dysfunction in the temporal dimension that may get affected by neurodegenerative diseases like AD where synaptic loss and dysfunction of network architecture of neural circuits are early features.^64^

Importantly, the current study establishes gamma PAC as a quantitative measure of neural circuit hyperexcitability in patients with AD. The classic method of detecting hyperexcitability in clinical populations is with the identification of epileptic activity in electrophysiological recordings via visual reads by experts. However, the subclinical manifestations of AD related neural circuit hyperexcitability put a high tab for resources for this approach as it requires long hours of recordings and reading of electrophysiological data,^65^ notwithstanding the fact that the results will only deliver a binary outcome. Quantitative measures of abnormal neurophysiological manifestations that are sensitive indices of neural circuit hyperexcitability are much needed tools in AD clinical populations, as these can be used in shorter recordings as well as to generate a graded index of hyperexcitability in each patient. The current study, being designed on the classic detection of spikes and sharps clearly demonstrates that gamma PAC is a robust and a sensitive tool that can track AD related hyperexcitability changes in local circuits. As this methodology can be applied to larger electrophysiological datasets collected from patients with AD, some of these being publicly available, future studies can be geared to refine and optimize gamma PAC as a widely applicable quantitative tool that can be useful for clinical trials.

### Limitations

Our findings should be considered in the context of the following limitations. Given the high demand on resources, LTM-EEG monitoring was only done for the AD patients and not for the controls. It is, however, important to establish the effect of ageing on network hyperexcitability. Our current patient cohort is predominantly early-onset AD phenotype and, therefore may not generalize to the more common late-onset AD population. Given the extreme demands on resources to identify subclinical epileptic events in electrophysiological recordings by expert readers, which we have set as the standard to categorically identify patients as AD-EPI+ and AD-EPI−, we have relied on the current cohort as a convenience sample, which was primarily collected as an observation study to investigate subclinical epileptic activity in AD and specifically focused on early onset and early stage AD cohort which were likely to manifest more of EPI+ phenotype.^66^ Notwithstanding this constraint, as our sample robustly represents the AD-EPI+ phenotype it facilitates the robust identification of quantitative metrics of hyperexcitability. The current sample size limits our ability to investigate whether reduced gamma PAC is different in male versus female sex, as well as whether it is affected in different ethnicities. Larger cohort studies with more representative samples will address these important issues. Given a hardware filter in the data-collection protocol set at 50 Hz, the current investigation focused on the gamma oscillations within the 30–40 Hz oscillatory window to avoid any filter associated artefacts. While the low gamma band represents the most commonly studied oscillatory window for gamma, future investigations are warranted to examine the PAC of high-gamma frequency range in AD.

## Supplementary Material

fcae121_Supplementary_Data

## Data Availability

All data and scripts associated with this study are present in the paper or in the Supplementary material. Anonymized subject data will be shared on request from qualified investigators for the purposes of replicating procedures and results, and for other non-commercial research purposes within the limits of participants’ consent. Correspondence and material requests should be addressed to the corresponding author.

## References

[1] Palop JJ , Chin J, Roberson ED, et al Aberrant excitatory neuronal activity and compensatory remodeling of inhibitory hippocampal circuits in mouse models of Alzheimer’s disease. Neuron. 2007;55(5):697–711. 17785178 10.1016/j.neuron.2007.07.025 PMC8055171

[2] Busche MA , Chen X, Henning HA, et al Critical role of soluble amyloid-beta for early hippocampal hyperactivity in a mouse model of Alzheimer’s disease. Proc Natl Acad Sci U S A. 2012;109(22):8740–8745. 22592800 10.1073/pnas.1206171109 PMC3365221

[3] Busche MA , Hyman BT. Synergy between amyloid-beta and tau in Alzheimer’s disease. Nat Neurosci. 2020;23(10):1183–1193. 32778792 10.1038/s41593-020-0687-6 PMC11831977

[4] Vossel KA , Ranasinghe KG, Beagle AJ, et al Incidence and impact of subclinical epileptiform activity in Alzheimer’s disease. Ann Neurol. 2016;80(6):858–870. 27696483 10.1002/ana.24794 PMC5177487

[5] Lam AD , Deck G, Goldman A, Eskandar EN, Noebels J, Cole AJ. Silent hippocampal seizures and spikes identified by foramen ovale electrodes in Alzheimer’s disease. Nat Med. 2017;23(6):678–680. 28459436 10.1038/nm.4330 PMC5461182

[6] Horvath AA , Papp A, Zsuffa J, et al Subclinical epileptiform activity accelerates the progression of Alzheimer’s disease: A long-term EEG study. Clin Neurophysiol. 2021;132(8): 1982–1989. 34034963 10.1016/j.clinph.2021.03.050

[7] Wu JW , Hussaini SA, Bastille IM, et al Neuronal activity enhances tau propagation and tau pathology in vivo. Nat Neurosci. 2016;19(8):1085–1092. 27322420 10.1038/nn.4328 PMC4961585

[8] Rodriguez GA , Barrett GM, Duff KE, Hussaini SA. Chemogenetic attenuation of neuronal activity in the entorhinal cortex reduces Abeta and tau pathology in the hippocampus. PLoS Biol. 2020;18(8):e3000851. 32822389 10.1371/journal.pbio.3000851 PMC7467290

[9] Buzsaki G , Wang XJ. Mechanisms of gamma oscillations. Annu Rev Neurosci. 2012;35(1):203–225. 22443509 10.1146/annurev-neuro-062111-150444 PMC4049541

[10] Palop JJ , Mucke L. Network abnormalities and interneuron dysfunction in Alzheimer disease. Nat Rev Neurosci. 2016;17(12):777–792. 27829687 10.1038/nrn.2016.141 PMC8162106

[11] Verret L , Mann EO, Hang GB, et al Inhibitory interneuron deficit links altered network activity and cognitive dysfunction in Alzheimer model. Cell. 2012;149(3):708–721. 22541439 10.1016/j.cell.2012.02.046 PMC3375906

[12] Jia X , Kohn A. Gamma rhythms in the brain. PLoS Biol. 2011;9(4):e1001045. 21556334 10.1371/journal.pbio.1001045 PMC3084194

[13] Tallon-Baudry C , Bertrand O. Oscillatory gamma activity in humans and its role in object representation. Trends Cogn Sci. 1999;3(4):151–162. 10322469 10.1016/s1364-6613(99)01299-1

[14] Fries P , Reynolds JH, Rorie AE, Desimone R. Modulation of oscillatory neuronal synchronization by selective visual attention. Science. 2001;291(5508):1560–1563. 11222864 10.1126/science.1055465

[15] Jensen O , Colgin LL. Cross-frequency coupling between neuronal oscillations. Trends Cogn Sci (Regul Ed). 2007;11(7):267–269. 10.1016/j.tics.2007.05.003 17548233

[16] Siebenhuhner F , Wang SH, Arnulfo G, et al Genuine cross-frequency coupling networks in human resting-state electrophysiological recordings. PLoS Biol. 2020;18(5):e3000685. 32374723 10.1371/journal.pbio.3000685 PMC7233600

[17] van der Meij R , Kahana M, Maris E. Phase-amplitude coupling in human electrocorticography is spatially distributed and phase diverse. J Neurosci. 2012;32(1):111–123. 22219274 10.1523/JNEUROSCI.4816-11.2012 PMC6621324

[18] Canolty RT , Edwards E, Dalal SS, et al High gamma power is phase-locked to theta oscillations in human neocortex. Science. 2006;313(5793):1626–1628. 16973878 10.1126/science.1128115 PMC2628289

[19] Lakatos P , Shah AS, Knuth KH, Ulbert I, Karmos G, Schroeder CE. An oscillatory hierarchy controlling neuronal excitability and stimulus processing in the auditory cortex. J Neurophysiol. 2005;94(3):1904–1911. 15901760 10.1152/jn.00263.2005

[20] Fries P . A mechanism for cognitive dynamics: Neuronal communication through neuronal coherence. Trends Cogn Sci. 2005;9(10):474–480. 16150631 10.1016/j.tics.2005.08.011

[21] Ranasinghe KG , Kudo K, Hinkley L, et al Neuronal synchrony abnormalities associated with subclinical epileptiform activity in early-onset Alzheimer’s disease. Brain. 2022;145(2):744–753. 34919638 10.1093/brain/awab442 PMC9630715

[22] Jack CR Jr , Bennett DA, Blennow K, et al NIA-AA Research framework: Toward a biological definition of Alzheimer’s disease. Alzheimers Dement. 2018;14(4):535–562. 29653606 10.1016/j.jalz.2018.02.018 PMC5958625

[23] McKhann GM , Knopman DS, Chertkow H, et al The diagnosis of dementia due to Alzheimer’s disease: Recommendations from the National Institute on Aging-Alzheimer’s Association workgroups on diagnostic guidelines for Alzheimer’s disease. Alzheimers Dement. 2011;7(3):263–269. 21514250 10.1016/j.jalz.2011.03.005 PMC3312024

[24] Cai C , Kang H, Kirsch HE, et al Comparison of DSSP and tSSS algorithms for removing artifacts from vagus nerve stimulators in magnetoencephalography data. J Neural Eng. 2019;16(6):066045. 31476752 10.1088/1741-2552/ab4065

[25] Dalal SS , Zumer JM, Guggisberg AG, et al MEG/EEG source reconstruction, statistical evaluation, and visualization with NUTMEG. Comput Intell Neurosci. 2011;2011:758973. 21437174 10.1155/2011/758973 PMC3061455

[26] Desikan RS , Segonne F, Fischl B, et al An automated labeling system for subdividing the human cerebral cortex on MRI scans into gyral based regions of interest. Neuroimage. 2006;31(3):968–980. 16530430 10.1016/j.neuroimage.2006.01.021

[27] Babiloni C , Arakaki X, Azami H, et al Measures of resting state EEG rhythms for clinical trials in Alzheimer’s disease: Recommendations of an expert panel. Alzheimers Dement. 2021;17(9):1528–1553. 33860614 10.1002/alz.12311 PMC8647863

[28] Ranasinghe KG , Cha J, Iaccarino L, et al Neurophysiological signatures in Alzheimer’s disease are distinctly associated with TAU, amyloid-beta accumulation, and cognitive decline. Sci Transl Med. 2020;12(534):eaaz4069. 32161102 10.1126/scitranslmed.aaz4069 PMC7138514

[29] Ranasinghe KG , Petersen C, Kudo K, et al Reduced synchrony in alpha oscillations during life predicts post mortem neurofibrillary tangle density in early-onset and atypical Alzheimer’s disease. Alzheimers Dement. 2021;17(12): 2009–2019. 33884753 10.1002/alz.12349 PMC8528895

[30] Pusil S , Lopez ME, Cuesta P, Bruna R, Pereda E, Maestu F. Hypersynchronization in mild cognitive impairment: The ‘X’ model. Brain. 2019;142(12):3936–3950. 31633176 10.1093/brain/awz320

[31] Canuet L , Pusil S, Lopez ME, et al Network disruption and cerebrospinal fluid amyloid-beta and phospho-Tau levels in mild cognitive impairment. J Neurosci. 2015;35(28):10325–10330. 26180207 10.1523/JNEUROSCI.0704-15.2015 PMC6605340

[32] Palva S , Palva JM. Functional roles of alpha-band phase synchronization in local and large-scale cortical networks. Front Psychol. 2011;2:204. 21922012 10.3389/fpsyg.2011.00204 PMC3166799

[33] Pontecorvo MJ , Devous MD, Kennedy I, et al A multicentre longitudinal study of flortaucipir (18F) in normal ageing, mild cognitive impairment and Alzheimer’s disease dementia. Brain. 2019;142(6):1723–1735. 31009046 10.1093/brain/awz090 PMC6536847

[34] Wang L , Benzinger TL, Su Y, et al Evaluation of Tau imaging in staging Alzheimer disease and revealing interactions between beta-amyloid and tauopathy. JAMA Neurol. 2016;73(9):1070–1077. 27454922 10.1001/jamaneurol.2016.2078 PMC5237382

[35] Jagust WJ , Landau SM. Alzheimer’s disease neuroimaging I. temporal dynamics of beta-amyloid accumulation in aging and Alzheimer disease. Neurology. 2021;96(9):e1347–e1357. 33408147 10.1212/WNL.0000000000011524 PMC8055327

[36] de Flores R , Das SR, Xie L, et al Medial temporal lobe networks in Alzheimer’s disease: Structural and molecular vulnerabilities. J Neurosci. 2022;42(10):2131–2141. 35086906 10.1523/JNEUROSCI.0949-21.2021 PMC8916768

[37] Chen X , Cassady KE, Adams JN, Harrison TM, Baker SL, Jagust WJ. Regional Tau effects on prospective cognitive change in cognitively normal older adults. J Neurosci. 2021;41(2):366–375. 33219003 10.1523/JNEUROSCI.2111-20.2020 PMC7810658

[38] Fu HJ , Possenti A, Freer R, et al A tau homeostasis signature is linked with the cellular and regional vulnerability of excitatory neurons to tau pathology. Nat Neurosci. 2019;22(1):47–56. 30559469 10.1038/s41593-018-0298-7 PMC6330709

[39] Voglein J , Ricard I, Noachtar S, et al Seizures in Alzheimer’s disease are highly recurrent and associated with a poor disease course. J Neurol. 2020;267(10):2941–2948. 32488295 10.1007/s00415-020-09937-7 PMC7501095

[40] Chang CW , Evans MD, Yu X, Yu GQ, Mucke L. Tau reduction affects excitatory and inhibitory neurons differently, reduces excitation/inhibition ratios, and counteracts network hypersynchrony. Cell Rep. 2021;37(3):109855. 34686344 10.1016/j.celrep.2021.109855 PMC8648275

[41] Lopez-Pigozzi D , Laurent F, Brotons-Mas JR, et al Altered oscillatory dynamics of CA1 parvalbumin basket cells during theta-gamma rhythmopathies of temporal lobe epilepsy. eNeuro. 2016;3(6):ENEURO.0284-16.2016. 10.1523/ENEURO.0284-16.2016 PMC5114702 27896315

[42] Sakkaki S , Barriere S, Bender AC, Scott RC, Lenck-Santini PP. Focal dorsal hippocampal Nav1.1 knock down alters place cell temporal coordination and spatial behavior. Cereb Cortex. 2020;30(9):5049–5066. 32377688 10.1093/cercor/bhaa101 PMC8475810

[43] Buzsaki G , Leung LW, Vanderwolf CH. Cellular bases of hippocampal EEG in the behaving rat. Brain Res. 1983;287(2):139–171. 6357356 10.1016/0165-0173(83)90037-1

[44] Fox SE , Wolfson S, Ranck JB Jr. Hippocampal theta rhythm and the firing of neurons in walking and urethane anesthetized rats. Exp Brain Res. 1986;62(3):495–508. 3720881 10.1007/BF00236028

[45] Pavlides C , Greenstein YJ, Grudman M, Winson J. Long-term potentiation in the dentate gyrus is induced preferentially on the positive phase of theta-rhythm. Brain Res. 1988;439(1–2):383–387. 3359196 10.1016/0006-8993(88)91499-0

[46] Poe GR , Nitz DA, McNaughton BL, Barnes CA. Experience-dependent phase-reversal of hippocampal neuron firing during REM sleep. Brain Res. 2000;855(1):176–180. 10650147 10.1016/s0006-8993(99)02310-0

[47] Iaccarino HF , Singer AC, Martorell AJ, et al Gamma frequency entrainment attenuates amyloid load and modifies microglia. Nature. 2016;540(7632):230–235. 27929004 10.1038/nature20587 PMC5656389

[48] Mably AJ , Gereke BJ, Jones DT, Colgin LL. Impairments in spatial representations and rhythmic coordination of place cells in the 3xTg mouse model of Alzheimer’s disease. Hippocampus. 2017;27(4):378–392. 28032686 10.1002/hipo.22697

[49] Mondragon-Rodriguez S , Gu N, Manseau F, Williams S. Alzheimer’s transgenic model is characterized by very early brain network alterations and beta-CTF fragment accumulation: Reversal by beta-secretase inhibition. Front Cell Neurosci. 2018;12:121. 29867356 10.3389/fncel.2018.00121 PMC5952042

[50] Stam CJ , van Cappellen van Walsum AM, Pijnenburg YA, et al Generalized synchronization of MEG recordings in Alzheimer’s disease: Evidence for involvement of the gamma band. J Clin Neurophysiol. 2002;19(6):562–574. 12488788 10.1097/00004691-200212000-00010

[51] Casula EP , Pellicciari MC, Bonni S, et al Decreased frontal gamma activity in Alzheimer disease patients. Ann Neurol. 2022;92(3):464–475. 35713198 10.1002/ana.26444 PMC9543336

[52] Rossini PM , Del Percio C, Pasqualetti P, et al Conversion from mild cognitive impairment to Alzheimer’s disease is predicted by sources and coherence of brain electroencephalography rhythms. Neuroscience. 2006;143(3):793–803. 17049178 10.1016/j.neuroscience.2006.08.049

[53] van Deursen JA , Vuurman EF, Verhey FR, van Kranen-Mastenbroek VH, Riedel WJ. Increased EEG gamma band activity in Alzheimer’s disease and mild cognitive impairment. J Neural Transm (Vienna). 2008;115(9):1301–1311. 18607528 10.1007/s00702-008-0083-y PMC2525849

[54] Ribary U , Ioannides AA, Singh KD, et al Magnetic field tomography of coherent thalamocortical 40-Hz oscillations in humans. Proc Natl Acad Sci U S A. 1991;88(24):11037–11041. 1763020 10.1073/pnas.88.24.11037 PMC53068

[55] Cuesta P , Ochoa-Urrea M, Funke M, et al Gamma band functional connectivity reduction in patients with amnestic mild cognitive impairment and epileptiform activity. Brain Commun. 2022;4(2):fcac012. 35282163 10.1093/braincomms/fcac012 PMC8914494

[56] Ossenkoppele R , Schonhaut DR, Scholl M, et al Tau PET patterns mirror clinical and neuroanatomical variability in Alzheimer’s disease. Brain. 2016;139(Pt 5):1551–1567. 26962052 10.1093/brain/aww027 PMC5006248

[57] Petersen C , Nolan AL, de Paula Franca Resende E, et al Alzheimer’s disease clinical variants show distinct regional patterns of neurofibrillary tangle accumulation. Acta Neuropathol. 2019;138(4):597–612. 31250152 10.1007/s00401-019-02036-6 PMC7012374

[58] Gorno-Tempini ML , Brambati SM, Ginex V, et al The logopenic/phonological variant of primary progressive aphasia. Neurology. 2008;71(16):1227–1234. 18633132 10.1212/01.wnl.0000320506.79811.da PMC2676989

[59] Buzsaki G . Rhythms of the brain. Oxford University press; 2011.

[60] McLardy T . Hippocampal formation of brain as detector-coder of temporal patterns of information. Perspect Biol Med. 1959;2(4):443–452. 13667392 10.1353/pbm.1959.0018

[61] Lisman JE , Idiart MA. Storage of 7 ± 2 short-term memories in oscillatory subcycles. Science. 1995;267(5203):1512–1515. 7878473 10.1126/science.7878473

[62] Jensen O . Maintenance of multiple working memory items by temporal segmentation. Neuroscience. 2006;139(1):237–249. 16337089 10.1016/j.neuroscience.2005.06.004

[63] Fries P . Rhythms for cognition: Communication through coherence. Neuron. 2015;88(1):220–235. 26447583 10.1016/j.neuron.2015.09.034 PMC4605134

[64] Sacks DD , Schwenn PE, McLoughlin LT, Lagopoulos J, Hermens DF. Phase–amplitude coupling, mental health and cognition: Implications for adolescence. Front Hum Neurosci. 2021;15:622313. 33841115 10.3389/fnhum.2021.622313 PMC8032979

[65] Vossel KA , Tartaglia MC, Nygaard HB, Zeman AZ, Miller BL. Epileptic activity in Alzheimer’s disease: Causes and clinical relevance. Lancet Neurol. 2017;16(4):311–322. 28327340 10.1016/S1474-4422(17)30044-3 PMC5973551

[66] Vossel KA , Beagle AJ, Rabinovici GD, et al Seizures and epileptiform activity in the early stages of Alzheimer disease. JAMA Neurol. 2013;70(9):1158–1166. 23835471 10.1001/jamaneurol.2013.136 PMC4013391

