# Supplementary material for: Abnormal gamma phase-amplitude coupling in the parahippocampal cortex is associated with network hyperexcitability in Alzheimer’s disease
Source: Brain Commun. 2024 Apr 9;6(2):fcae121. doi: 10.1093/braincomms/fcae121 (PMC11043655; doi:10.1093/braincomms/fcae121)
Supplement: fcae121_Supplementary_Data [file fcae121_supplementary_data.zip › Original Submission.pdf]

OXFORD UNIVERSITY PRESS | Brain Communications

Abnormal Gamma Phase-Amplitude Coupling in the Parahippocampal Cortex is Associated with Network Hyperexcitability in Alzheimer's Disease

Journal:	*Brain Communications*
Manuscript ID	BRAINCOM-2023-534
Manuscript Type:	Original Article
Date Submitted by the Author:	22-Nov-2023
Complete List of Authors:	Prabhu, Pooja; University of California San Francisco, Department of Radiology and Biomedical Imaging; Manipal Institute of Technology, Department of Data science and Computer Applications<br>Morise, Hirofumi; Medical Imaging Center, Ricoh Company, Ltd; Ricoh Company, Ltd., Medical Imaging Business Center<br>Kudo, Kiwamu; University of California San Francisco, Department of Radiology and Biomedical Imaging; Ricoh Company, Ltd., Medical Imaging Business Center<br>Beagle, Alexander; UC San Francisco, Memory and Aging Center<br>Mizuiri, Danielle; University of California, San Francisco, Radiology and Biomedical Imaging<br>Syed, Faatimah; University of California San Francisco<br>Kotegar, Karunakar A.; Manipal Institute of Technology, Department of Data science and Computer Applications<br>Findlay, Anne; University of California San Francisco, Department of Radiology and Biomedical Imaging<br>Miller, Bruce; University of California San Fransisco , Memory and Aging Center, Department of Neurology<br>Kramer, Joel; University of California San Fransisco , Memory and Aging Center, Department of Neurology<br>Rankin, Katherine; University of California San Fransisco , Memory and Aging Center, Department of Neurology<br>Garcia, Paul; University of California San Francisco, Department of Neurology<br>Kirsch, Heidi; University of California, San Francisco, Department of Neurology<br>Vossel, Keith; University of California Los Angeles; University of California San Francisco, Memory and Aging Center, Department of Neurology<br>Nagarajan, Srikantan; UCSF, Department of Radiology and Biomedical Imaging<br>Ranasinghe, Kamalini; University of California San Francisco, Memory and Aging Center, Department of Neurology
Keywords:	phase amplitude coupling, gamma oscillations, network hyperexcitability, Alzheimer's disease, Magnetoencephalography, Parahippocampus

SCHOLARONE™
Manuscripts

Abnormal Gamma Phase-Amplitude Coupling in the Parahippocampal Cortex is Associated with Network Hyperexcitability in Alzheimer's Disease

Pooja Prabhu[1,2], Hirofumi Morise[1,3], Kiwamu Kudo[1,3], Alexander Beagle[4], Danielle Mizuiri[1], Faatimah Syed[4], Karunakar A. Kotegar[2], Anne Findlay[1], Bruce L. Miller[4], Joel H. Kramer[4], Katherine P. Rankin[4], Paul A. Garcia[5], Heidi E. Kirsch[1,5], Keith Vossel[4,6], Srikantan S. Nagarajan[1], Kamalini G. Ranasinghe[4]*

[1] Department of Radiology and Biomedical Imaging, University of California San Francisco, San Francisco, CA, USA 94158

[2] Department of Data science and Computer Applications, Manipal Institute of Technology, Manipal, India

[3] Medical Imaging Business Center, Ricoh Company Ltd., Kanazawa, Japan

[4] Memory and Aging Center, Department of Neurology, University of California San Francisco, San Francisco, CA, USA 94158

[5] Epilepsy Center, Department of Neurology, University of California San Francisco, San Francisco, CA, USA 94158

[6] Mary S. Easton Center for Alzheimer's Research and Care, Department of Neurology, David Geffen School of Medicine, University of California Los Angeles, Los Angeles, CA 90095

Corresponding author: Kamalini G Ranasinghe

Memory and Aging Center, Department of Neurology

University of California San Francisco

675 Nelson Rising Lane, Suite 190, San Francisco, CA 94158-1207

Tel: 415-514-8847

e-mail: Kamalini.ranasinghe@ucsf.edu

For Review Only

ABSTRACT

While animal models of Alzheimer's disease (AD) have shown altered gamma oscillations (~40 Hz) in local neural circuits, the low signal-to-noise ratio of gamma in the resting human brain preclude its quantification via conventional spectral estimates. Phase-amplitude coupling (PAC) indicating the dynamic integration between the gamma amplitude and the phase of low frequency (4-12 Hz) oscillations is a useful alternative to capture the local gamma activity. In addition, PAC is also an index of neuronal excitability as the phase of low frequency oscillations that modulate the amplitude of gamma, effectively regulate the excitability of local neuronal firing. In this study, we sought to examine the local neuronal activity and excitability using gamma PAC, within brain regions vulnerable to early AD pathophysiology—entorhinal cortex and parahippocampus, in a clinical population of patients with AD and age-matched controls. Our clinical cohorts consisted of a well-characterized cohort of AD patients (n=50; age, 60±8 years) with positive AD biomarkers, and age-matched cognitively unimpaired controls (n=35; age, 63±5.8 years). We identified the presence or absence of epileptiform activity in AD patients (AD-EPI+, n=20; AD-EPI−, n=30) using long-term electroencephalography (LTM-EEG) and 1-hour long magnetoencephalography (MEG) with simultaneous EEG. Using the source reconstructed MEG data, we computed gamma PAC as the coupling between amplitude of the gamma frequency (30-40 Hz) with phase of the theta (4-8 Hz) and alpha (8-12 Hz) frequency oscillations, within entorhinal and parahippocampal cortices. We found that patients with AD have reduced gamma PAC in the left parahippocampal cortex, compared to age-matched controls. Furthermore, AD-EPI+ patients showed greater reductions in gamma PAC than AD-EPI− in bilateral parahippocampal cortices. In contrast, entorhinal cortices did not show gamma PAC differences either between AD versus control or between AD-EPI− versus AD-EPI+. Our findings demonstrate the specific regional patterns of altered gamma oscillations within medial temporal cortex regions vulnerable to AD pathophysiology indicating possible region-specific vulnerabilities of network hyperexcitability.

For Review Only

Greater deficits in AD-EPI+ suggested that reduced gamma PAC is a sensitive index of network hyperexcitability in AD patients. Collectively, the current results emphasize the importance of investigating the role of neural circuit hyperexcitability in early AD pathophysiology and explore its potential as a modifiable contributor to AD pathobiology.

For Review Only

1. Introduction

Animal models of Alzheimer's disease (AD) have demonstrated that network hyperexcitability is strongly associated with AD proteinopathy and behavioural deficits[1,2]. These studies have identified that aberrant neuronal firing is an early phenomenon in AD pathogenesis[3]. Consistent with these basic science evidence suggesting network hyperexcitability is closely associated with disease pathophysiology, clinical studies in patients have also demonstrated high incidence of epileptic manifestations, either as overt seizures or as subclinical epileptiform discharges in patients with AD[4-6]. Importantly, neural circuit hyperexcitability is a key mechanism that contribute to AD pathophysiology within regions that are vulnerable to AD-tauopathy[7]. For example, a transgenic mouse model which overexpressed both human amyloid precursors protein (hAPP) and entorhinal tau showed that amyloid-beta (Aβ) significantly increased the excitability of entorhinal neurons which then facilitated the spread of tau into the downstream hippocampus while attenuation of the hyperexcitability reduced these pathologies[8]. Patterns of altered local excitability in the medial temporal lobes in patients with AD remains largely unknown.

While neural oscillations represent the composite activity of excitatory firing of principal cells and rhythmic inhibition of interneurons arranged into hierarchical cellular networks, local excitability is thought to be best represented in the gamma range frequency (>30 Hz)[9]. Gamma abnormalities are indeed the the most frequently reported oscillatory abnormalities in AD transgenic mice[10,11]. However, in the human brain, a major obstacle in examining gamma band activity from non-invasive neuroimaging is its low signal-to-noise ratio due to the distance of sensors from the cortex and contamination from other muscular activity artifacts[12]. While increased gamma power is observed in a region-dependent manner when engaged in a cognitive task[13,14], during rest it reaches to very low amplitudes, almost undetectable by standard power density assays. In this context, an effective means to capture gamma activity, even at rest, is cross-frequency coupling

https://mc.manuscriptcentral.com/braincom

between gamma oscillations and lower frequency theta and alpha oscillations[15,16]. Phase amplitude coupling (PAC), which specifies the statistical dependence between the amplitude of a high-frequency oscillation (i.e., gamma)[17,18], with the phase of a low-frequency (i.e., theta or alpha) oscillation and is of particular interest here because by modulating the amplitude of the high frequency, the phase of the low frequency oscillations essentially regulate the excitability of local neuronal firing[19,20]. Quantification of gamma band cross-frequency-coupling, therefore, not only provides a measure of local neuronal activity but also an index of neuronal excitability within an ensemble regional network.

In this study, we sought to examine the local neuronal activity and hyperexcitability using gamma PAC within brain regions vulnerable to early AD pathophysiology—entorhinal cortex and parahippocampus. Specifically, we examined the coupling between amplitude of the gamma frequency (30-40 Hz) with phase of the theta (4-8 Hz) and alpha (8-12 Hz) frequency oscillations. We categorically identified AD patients with and without epileptiform activity—the cardinal clinical manifestations of network hyperexcitability, and examined the theta-gamma and alpha-gamma PAC in patients with and without subclinical epileptiform activity (AD-EPI+ vs. AD-EPI−). We hypothesized that abnormal gamma PAC would indicate relative patterns of network hyperexcitability within the medial temporal lobes of entorhinal and parahippocampal cortices associated with AD pathophysiology.

2. Materials and Methods

2.1. Participants

We used a well-characterized cohort of AD patients that was described in a previous investigation[21] (n=50 AD patients; age, 60±8 years) and thirty-five age-matched controls (age, 63±5.8 years). All patients met National Institute of Aging-Alzheimer's Association criteria with positive biomarkers, including cerebrospinal fluid (CSF) and/or positive amyloid reading in

For Review Only

positron emission tomography (PET), or histopathological confirmation at autopsy (Supplementary material) [22,23]. Out of 50 AD patients, 20 patients had subclinical epileptiform activity (AD-EPI+), and the remaining 30 did not show epileptiform activity (AD-EPI−), in long-term monitoring by video EEG (LTM-EEG) and magnetoencephalography (MEG) evaluations. Age-matched controls were recruited based on the criteria: normal cognitive performance; normal magnetic resonance imaging (MRI); and absence of neurological, psychiatric, or other major illnesses. All the participants were recruited through the University of California San Francisco (UCSF) Memory and Aging Center. Informed consent was obtained from each participant or their surrogate decision makers. The study was approved by the UCSF Institutional Review Board.

2.2. Data acquisition

Each patient underwent overnight LTM-EEG, which was recorded using silver cup electrodes placed in 10-20 electrode array, with three minutes of hyperventilation. LTM-EEG was acquired at the Clinical and Translational Science Institute Clinical Research Center at Moffitt Hospital at UCSF. On the following day patients underwent one hour resting-state MEG recording and simultaneous 21-lead EEG recording. Whole-head CTF MEG system with 275 axial gradiometers was used (sampling rate of 600 Hz). To co-register MEG with brain MRI and to obtain a head position with respect to the sensor array, fiducial coils were placed at nasion, left pre-auricular and right pre-auricular points. Participants were in a supine position with their eyes closed during the MEG acquisition. Out of 35 controls, 17 were monitored based on LTM-EEG and M/EEG; 8 were monitored based on 1-hour resting-state M/EEG; and 10 were monitored with 10-minutes of resting-state MEG. None of the control participants showed no evidence of epileptiform manifestations within their respective electrophysiological recordings. Epileptiform discharges were identified using the LTM-EEG and M/EEG in AD patients as described previously[4,21]. All participants structural MRI were acquired and then used to generate individualized head models for MEG source reconstruction.

For Review Only

2.3. Source Reconstruction of MEG data

We selected a contiguous 60-second data epoch from the initial segment of the awake resting MEG recording. The awake-resting state of each participant was further assured by inspecting the power spectral density of the 60-second dataset. Artefact detection was based on visual inspection of channels and trials, ensuring that the peak magnetic field of the data did not cross 1pT. Data that was beyond this threshold was subjected to established denoising algorithms[24]. Spatiotemporal estimates of neural sources were generated using time-frequency analyses implemented in the Neurodynamic Utility Toolbox for MEG (NUTMEG; http://nutmeg.berkeley.edu)[25]. Source-space reconstructions of MEG sensor data were generated using a Linear-Constrained Minimum Variance (LCMV) beamformer with a 10 mm lead field. Voxel-based source signals were mapped to the Desikan-Killany (DK) atlas[26] to obtain regional time series (Figure 1A). To test our main hypothesis, we used *apriori* selected, known medial temporal lobe regions that show highest vulnerability to early AD pathophysiology. In the DK atlas parcellations these regions included the right and left entorhinal cortices, and right and left parahippocampal cortices.

2.4. Phase-Amplitude Coupling

We estimated PAC using coupling strength (via mean vector length) between the phase of lower frequency (LF) oscillation (4-12 Hz) and amplitude of high frequency (HF) oscillation (30-40 Hz) for every 1 Hz frequency bin (Figure 1B). Specifically, phase and amplitude pairs are generated for a grid of 80 PAC combinations. For each of four region's time series, we computed the modulation index[18] between the phase of theta (4-8Hz) or alpha (8-12 Hz) and the amplitude of the gamma (30-40 Hz). The modulation index was computed as the complex-valued composite signal, which is the combination of amplitude time series (30-40 Hz) with the phase time series (4-12 Hz). The amplitude time series ($A_H(t)$) was obtained by extracting the envelope (or amplitude) by applying Hilbert transform to a high frequency bandpass signal (30-40 Hz). The

phase time series ($\Phi_L(t)$) was obtained by extracting the phase by applying Hilbert transform to low frequency bandpass signal (4-12 Hz). The complex-valued composite signal is Z(t) = A_H(t) $e^{i\Phi_L(t)}$. The coupling between the A_H and Φ_L is the degree of asymmetry of the probability density function of Z(t) which was obtained by measuring the mean of Z(t) (denoted by M_{raw}). The mean of Z(t) was first normalized to examine the joint distribution of A_H and Φ_L by considering the forms of the marginal distribution of A_H alone and Φ_L alone. To accomplish this, we compared the mean with the set of means from surrogates (n=200). Surrogates were generated by introducing time lag τ such that Z(t, τ) = A_H(t + τ) $e^{i\Phi_L(t)}$. For large τ, the asymmetry in Z(t, τ) in distribution was due to Φ_L and A_H alone determined how far points fall from the origin. The normalised (or z-scored) mean was M_{norm}= (|M_{raw}|-μ)/σ, where μ and σ is the mean and standard deviation of the surrogate's length, respectively. The normalised phase angle, which defines the temporal relationship between the low frequency and the gamma signal (Φ_{norm}) was computed from the complex-value M_{raw}. Thus, the coupling strength between the time series of two frequency bands was indicated as M_{norm} and the phase angle between them was indicated as Φ_{norm}. Idealized depiction of high coupling and low coupling is shown in Figure 1B. We generated polar plots where each point corresponds to complex values of the composite signal (Figure 1B). To compute PAC from the regional time series, we used the EEGlab event-related PAC toolbox (https://github.com/sccn/PACTools).

2.5. Statistical Analysis

We examined the group differences between AD and age-matched controls, and AD-EPI+ and AD-EPI−. To correct for false positives arising from multiple comparisons in the phase-amplitude grid (80 elements) per region, we used a permutation-based cluster test. Independent t-tests were calculated for every PAC value on the grid at the region-level between groups. T-values that were adjacent in both phase and amplitude frequency below a cluster alpha threshold of 5% were added up to create clusters. After 1024 permutations (or repetitions) of randomly alternating PAC

For Review Only

values within each region, a permutation distribution was calculated. By contrasting the cluster statistic with the random permutation distribution, the permutation p-value was found. When the total of the t-values was more than 95% (or $p<0.05$) of the permuted distribution, the observed clusters were regarded as statistically significant. We also calculated the mean phase angle as the average across the PAC-grid combinations that showed group difference in mean vector length between group contrasts (i.e. AD vs. age-matched controls and AD-EPI− vs. AD-EPI+), for each subject. The mean phase angle was obtained by averaging the complex values and then calculating the angle of the resultant complex value. We also compared the phase angle of the PAC-grid combinations that showed group difference in mean vector length between group contrasts, using unpaired t-tests.

For Review Only

3. Results

3.1. Participant demographics

Clinical and demographic characteristics of AD patients and controls used in the current study have been detailed in a previous report[21] and also provided in the Supplementary table 1. AD patients with and without epileptiform activity were matched in their age and other demographics (AD-EPI+: age, 59.9 ± 6.7 years, female sex, 12 (60%), education, 17 ± 2.7 years, and AD-EPI−: age, 60.7 ± 8.3 years, female sex, 17 (56.7%), education, 15.7 ± 2.6 years), as well as in clinical and cognitive characteristics (Supplementary table 2).

3.2. Reduced theta-gamma coupling in the left parahippocampal cortex in AD

The patterns of gamma PAC within the parahippocampal cortices showed reductions in patients with AD, especially with in the left parahippocampus, compared to age-matched controls (Fig. 2A-B), while the entorhinal gamma PAC values seems similar across both groups (Fig. 2C-D). Specifically, in the left parahippocampal cortex, patients with AD showed reduced theta-gamma PAC compared to age-matched controls, where gamma amplitude showed reduced coupling

within the 6-8 Hz oscillatory range (Fig. 3A-B). The average normalized PAC of gamma amplitudes that showed reduced coupling for the 6-8 Hz phase at cohort level was -0.235 for AD patients and 0.312 for controls (Fig. 3A, P=0.037). The mean phase angle was computed as the average across the PAC-grid combinations that showed group difference between AD and controls, for each subject's left parahippocampal region. The mean phase angle (dotted line in Fig. 3B) in patients with AD was $122^{\circ,}$ preceding the phase angle in age-matched controls at 232° (Fig. 3B). Although not significantly different from each other, both groups were closer to 90° or 270° than 0° or 180° phase (t=0.3984, P=0.6914). In summary, the coupling strength in the left parahippocampus is significantly reduced while the timing of neuronal activity patterns that contribute to PAC are not significantly altered in AD.

3.3. Reduced gamma PAC in bilateral parahippocampal cortices in AD-EPI+

Parahippocampal cortices showed greater reductions in AD-EPI+ compared to AD-EPI−, while entorhinal cortices in both groups showed similar patterns (Fig. 4). Specifically, AD-EPI+ showed reduced gamma PAC in the left parahippocampus (Fig. 5A-B, P=0.039) as well as in the right parahippocampus (Fig.5 C-D, P=0.008), compared to AD-EPI−. In particular, within the left parahippocampus, the gamma amplitudes showed reduced coupling with the phase of 4-7Hz range oscillations indicating reduced theta-gamma PAC (Fig. 5A-B), whereas in the right parahippocampus the gamma amplitude showed reduced coupling with the phase of 8-12Hz range of oscillations indicating reduced alpha-gamma PAC (Fig. 5C-D). Average phase angles were computed across the range of PAC-grid combinations that showed significant group differences between EPI+ and EPI− patients within each respective anatomical regions (i.e. theta-gamma PAC grid combinations in left parahippocampus; alpha-gamma PAC grid combinations in right parahippocampus). The mean phase angle (yellow dotted line in Fig. 5B and Fig. 5D) in AD-EPI− was at 96° and at 11° in the left and right parahippocampus, respectively . The mean phase angle of AD-EPI+ (red dotted line in Fig. 5B and Fig. 5D) although lagged the phase angle of AD-

For Review Only

EPI− in both left and right para-hippocampi showing 187° and 58°, respectively, these differences were not statistically significant (Left para hippocampus: t=-0.6742, p=0.5034; Right para hippocampus: t=0.5402, p=0.5916).

4. Discussion

In this study, we examined the local neuronal activity within gamma oscillations by quantifying the coupling between gamma amplitude and the phase of theta and alpha oscillations, in a well-characterized cohort of AD patients and age-matched controls. We found that patients with AD have reduced gamma coupling compared to controls in the left parahippocampus while AD-EPI+ patients showed greater reductions in gamma coupling within bilateral parahippocampal cortices compared to AD-EPI−. In contrast to parahippocampal cortices, entorhinal cortex did not show altered gamma oscillations either in AD vs. controls or in AD-EPI+ vs. AD-EPI−. Collectively our findings demonstrate: (1) greater gamma PAC deficits in AD-EPI+ suggesting that gamma PAC is a sensitive index of network hyperexcitability in AD; (2) region-specific alterations in gamma PAC suggesting regional vulnerabilities of network hyperexcitability within the medial temporal lobes in AD.

4.1. Hyperexcitability within medial temporal lobe subregions in AD

The current study demonstrated altered gamma PAC in patients with AD within parahippocampus but not within entorhinal cortex, compared to age-matched controls. This regional selectivity within medial temporal subregions is particularly intriguing given the regional specificity of age related tauopathy vs. aging related tauopathy[27,28]. The emergence of tau-PET has demonstrated the patterns of progressive tau accumulation in healthy aging and in individuals who have Aβ accumulation in the brain. These studies show that while entorhinal tau accumulation is aging related, spread of tau beyond entorhinal cortex and into the adjacent parahippocampal regions only happens in the presence of Aβ indicating AD neuropathological changes[29-31]. In this context,

For Review Only

altered hyperexcitability in AD patients in the parahippocampus but not in the entorhinal cortex compared to healthy aging pose the question whether neuronal hyperexcitability play a role in the progression of AD-tauopathy beyond entorhinal regions. Indeed, animal models have shown compelling evidence that locally increased neuronal activity stimulates the release of tau and enhances tau pathology[7,8,32]. The current study also demonstrated that patients with AD-EPI+ have greater reductions of gamma PAC involving bilateral parahippocampal cortices, suggesting a possible contributory role of hyperexcitability to the faster rate and more severe cognitive loss associated with AD-EPI+ phenotype[4,33]. While the relationships between regional patterns of medial temporal lobe hyperexcitability and AD-tauopathy is yet to be demonstrated in human neuroimaging studies, the current results emphasize the importance of investigating the role of neural circuit hyperexcitability in early AD pathophysiology and explore its potential as a modifiable contributor.

4.2. PAC and hyperexcitability in AD

Investigations in AD transgenic mice demonstrate that increased seizure susceptibility and network hyperexcitability are associated with early amyloid and tau accumulations[10,34]. Consistent with such evidence, we have previously demonstrated an increased incidence of subclinical epileptiform activity in patients with AD[4] and that AD-EPI+ patients have greater reductions in alpha and greater increases in delta-theta neuronal synchrony than age-matched controls[21]. The mechanistic relationships between these low frequency oscillatory abnormalities and network hyperexcitability, however, still remains incompletely understood. In contrast, many basic science epilepsy models and AD models have explored the relationship between the coupling of low frequency phase to gamma amplitude and epileptogenic phenomenon in local neural circuits. The current results showing reduced gamma coupling in patients with AD and the greater degree of such deficits in AD-EPI+ brings a step closer in understanding the mechanistic relationship between network hyperexcitability and neural oscillatory changes. A rodent model of temporal

lobe epilepsy (TLE) demonstrated reduced theta-gamma coupling in the dorsal hippocampus in TLE rats during the interictal period and that impaired inhibitory activity of parvalbumin (PV) basket cells as the major contributory cause for this rhythmopathy[35]. A mouse model with focal knock-down of SCN1A gene, which encode the Nav1.1 voltage-gated sodium channel in the dorsal hippocampus demonstrated reduced theta-gamma coupling in hippocampus[36]. In particular the Nav1.1 knock-down mice showed abnormal spatial memory performance and affected both pyramidal neuronal firing as well as fast-spiking inhibitory interneurons. Indeed abnormal Nav1.1 activity in PV basket cells have been identified as one of the possible contributory causes to network hyperexcitability in transgenic AD mice[11]. While much of the details of cellular and molecular details of these relationships are yet to be identified, the current results clearly emphasize that abnormal gamma PAC is a key manifestations of altered neural syntax closely coupled to epileptogenic processes associated with AD pathophysiology. Diminished gamma coupling physiologically may have clinical influences on memory and other cognitive functions in AD populations.

Compared to the strength of coupling in basic science disease models, much less is known about the phase angle of coupling in PAC. Under physiological conditions, however, compelling evidence support distinct roles of neuronal activity that occur during the peak and the trough of low frequency phase modulations such as theta. For example, in awake active rodents hippocampal place cell firing is actively coupled to the peak of the theta phase[37]. It has been shown that maximum depolarization of place cells occur at the peak of the theta cycle which facilitate long-term-potentiation, whereas firing in the theta trough, where depolarization is not optimized induce long-term-depression, in hippocampal circuits[38-40]. Although, theta-gamma coupling in resting-state brain may have its own physiological significance from the behaviourally active brain, the phase angle of coupling is still likely to play an important role. Although the phase angles were not significantly different between patients with AD and controls in the select regional

analysis of gamma PAC in our investigation, we believe this is an important area that warrants further investigation in AD. It is likely that the altered timing of neural activity may be associated with AD pathobiology in general, although not necessarily a metric that is indexing hyperexcitability in AD.

4.3. Gamma oscillatory changes in AD

Abnormal spectral signatures of increased delta-theta oscillatory activity and reduced alpha and beta oscillatory activity are well documented in clinical AD populations[41,42]. Abnormalities in the gamma band oscillatory activity, in contrast, have been difficult to capture, owing to its low signal-to-noise ratio in the spectral power density assay of the resting human brain. In transgenic AD mice, gamma oscillatory activity is consistently reduced[11,43] including reduced PAC of theta phase and slow gamma power[44,45]. Findings from human imaging studies show variable results. In one study, widespread reductions in gamma synchronization in patients with AD were reported from whole brain MEG sensor-space analysis[46], while the region of interest-based evaluation of frontal/prefrontal cortices using combined transcranial magnetic stimulation and EEG found reduced gamma power in patients with AD[47]. On the other hand, high midline gamma coherence in EEG was associated with a greater incidence of conversion from mild cognitive imapairment (MCI) to AD[48], while greater 40Hz steady-state activity in EEG was found in AD compared to MCI[49]. Apart from methodological differences, these latter studies could have been influenced by limited spatial resolution and poor source localization ability of EEG local field potential signals. Our findings showing reduced theta/alpha-gamma coupling delivered through high spatial resolution MEG source localisation techniques are consistent with previous MEG findings[46,50] as well as the collective findings from AD transgenic mice studies reporting reduced gamma oscillatory activity in AD. A recent MEG clinical investigation on patients diagnosed with MCI also showed that gamma band functional connectivity in the right temporal cortex was significantly reduced in those who were positive for epileptiform activity than those without[51]. It is likely that

gamma oscillatory activity, which represent the collective firing of local neuronal ensembles are sensitive to the spatial resolution of the imaging modality. Nonetheless, both basic and clinical science investigations provide the collective insight that activity within local circuits are significantly altered in AD, while the current study extend these insights indicating their potential mechanistic links to network hyperexcitability in AD.

4.4. PAC as a measure of local network dysfunction

Synchronized neural oscillations have been proposed as an effective mechanism of network communication in the brain[52]. While low-frequency oscillations, like theta, are specially adapted for long distance synchronizations, faster oscillations, such as gamma, represent ensemble neuronal activity over comparatively small spatial scales[15]. When moving from a local to a global scale, interactions between low and high frequency oscillations provide a mechanism to integrate these neural processes along the spatial hierarchy of brain activity[15]. When gamma oscillations appear during a specific phase cycle of the low frequency oscillation, attracting groups of cells actively engaged at that moment, a synchronized low frequency oscillation will help to combine these spatially distributed, locally functional activity patterns. It has also been suggested that different low frequency phases coupled to gamma oscillations may encode distinct information[53]. In this scheme, the cycle of lower oscillation can be divided into time slots and the coupled gamma oscillatory activity within each epoch encodes distinct representations of local neuronal activity[54,55]. PAC, which links the phase of the low frequency neural oscillation with the amplitude of the faster neuronal oscillation has been shown to be a highly reliable index and not only facilitates the separate spatially distributed cortical networks operating in parallel[17], but also provides the explanation of "neuronal communication through neuronal coherence"[56]. PAC, therefore, is an attractive metric to quantify a unique aspect of network dysfunction in the temporal dimension that may get affected by neurodegenerative diseases like AD where synaptic loss and dysfunction of network architecture of neural circuits are early features[57].

https://mc.manuscriptcentral.com/braincom

For Review Only

4.5. Limitations

Our findings should be considered in the context of the following limitations. Given the high demand on resources, LTM-EEG monitoring was only done for the AD patients and not for the controls. It is, however, important to establish the effect of aging on network hyperexcitability. Our current patient cohort is predominantly early-onset AD phenotype and, therefore may not generalize to the more common late-onset AD population. Given a hardware filter in the data-collection protocol set at 50Hz, the current investigation focused on the gamma oscillations within the 30-40 Hz oscillatory window to avoid any filter associated artefacts. While the low gamma band represents the most commonly studied oscillatory window for gamma, future investigations are warranted to examine the PAC of high-gamma frequency range in AD.

For Review Only

FIGURE LEGENDS

Figure 1: **Estimation of gamma band PAC**. MEG source-reconstructed region-level time series is mapped onto atlas parcellations to obtain time series for each cortical region of L and R entorhinal and L and R parahippocampal cortex. Each region-level time series was filtered at 4-12 Hz and 30-40 Hz to get phase and amplitude signals, respectively. Then, the coupling strength is measured using the mean vector length between the phase of 4-12 Hz signal and the amplitude of 30-40 Hz signal (B). Idealised depiction of high coupling and low coupling is shown in blue-to-white color gradient on the PAC grid. Polar plots corresponds to data points of complexed composite signals for the low and high PAC identified on the grid. Mean of these points (mean vector length) is represented in the green line. High coupling is represented by a large mean vector length and low coupling is represented by a small mean vector length. (Abbreviations: PAC, phase amplitude coupling)

Figure 2: **Gamma band PAC in patients with AD and age-matched controls in parahippocampal and entorhinal cortices**. Gamma PAC within parahippocampal cortices, especially the left-parahippocampus showed reductions in patients with AD than controls (A-B). Gamma PAC within entorhinal cortices showed similar patterns in age-matched controls and in patients with AD and (C-D). (Abbreviations: AD, Alzheimer's disease; PAC, phase amplitude coupling)

Figure 3: **Patients with AD showed reduced gamma PAC within left parahippocampus**. Patients with AD showed reduced coupling of gamma amplitude and theta (6-8 Hz) phase in the left parahippocampal cortex compared to age-matched controls (A). The blue-colored phase-amplitude coupling range showed statistically significant reductions in AD patients vs. age-

For Review Only

matched controls in the left parahippocampus. The phase angle distribution in the left parahippocampal region, within the significant phase-amplitude coupling range (as indicated in blue in subplot A), showed that AD patients (orange dotted line; mean phase angle=121.7^{o}) are leading age-matched controls (purple dotted line; mean phase angle=231.8^{o}) (B). (Abbreviations: AD, Alzheimer's disease; PAC, phase amplitude coupling)

Figure 4: **Gamma band PAC in AD-EPI− and AD-EPI+ in parahippocampal and entorhinal cortices**. Gamma PAC within both left and right parahippocampal cortices, showed reductions in patients with AD-EPI+ compared to patients with AD-EPI− (A-B). Gamma PAC within entorhinal cortices showed similar patterns in AD-EPI− and AD-EPI+ (C-D). (Abbreviations: AD, Alzheimer's disease; PAC, phase amplitude coupling)

Figure 5: **Patients with AD-EPI+ showed reduced gamma PAC in bilateral parahippocampal cortices compared to AD-EPI−**. Patients with AD-EPI+ showed reduced theta-gamma PAC in the left parahippocampal region (A). The blue-colored phase-amplitude coupling range showed statistically reductions in AD-EPI+ patients vs. AD-EPI− patients in the left hippocampus (B). The phase angle distribution in the left parahippocampal region, within the significant phase-amplitude coupling range (as indicated in blue in subplot B), showed that AD-EPI− (yellow dotted line; mean phase angle=95.8^{o}) are leading AD-EPI+ patients (red dotted line; mean phase angle=186.7^{o}). Patients with AD-EPI+ showed reduced alpha-gamma PAC in the right parahippocampal cortex (C). The phase angle distribution in the right parahippocampal region, within the significant phase-amplitude coupling range (as indicated in blue in subplot C), showed that AD-EPI− (yellow dotted line; mean phase angle=11.5^{o}) are leading AD-EPI+ patients (red dotted line; mean phase

angle=57.8°; D). (Abbreviations: AD, Alzheimer's disease; AD-EPI+, AD patients with epileptiform activity; AD-EPI−, AD patients without epileptiform activity; PAC, phase amplitude coupling).

For Review Only

ACKNOWLEDGMENTS: We would like to thank all of the study participants and their families for their generous support to our research. The preliminary results of the study were presented as poster in Cognitive Neuroscience Society (CNS), 2022 at San Francisco and in Alzheimer's Association International Conference (AAIC), 2022 at San Diego and Society of Neuroscience (SFN), 2022 at San Diego.

CONFLICTS OF INTEREST: The authors of this manuscript do not have any conflicts of interest relevant to the content of this work.

DATA AVAILABILITY: All data associated with this study are present in the paper or in the Supplementary material. Anonymized subject data will be shared on request from qualified investigators for the purposes of replicating procedures and results, and for other non-commercial research purposes within the limits of participants' consent. Correspondence and material requests should be addressed to the corresponding author.

FUNDING: This study was supported by the National Institutes of Health grants: K08AG058749 (K.G.R.), R21AG077498-01 (K.G.R), K23 AG038357 (K.V.), P50-AG023501 (B.L.M.) R01NS100440 (S.S.N.), R01DC017091 (S.S.N.), R01AG062196 (S.S.N.); a research contract with Ricoh MEG Inc. (S.S.N.); a grant from John Douglas French Alzheimer's Foundation (K.V.); the S.D. Bechtel Jr. Foundation (K.V.); grants from Larry L. Hillblom Foundation: 2015-A-034-FEL (K.G.R.) and 2019-A-013-SUP (K.G.R.); grants from the Alzheimer's Association: AARG-21-849773 (K.G.R.), PCTRB-13-288476-made possible by Part the CloudTM ETAC-09-133596 (K.V.), a doctoral fellowship by Fulbright-Nehru Doctoral Research Fellowship (P.P).

CONSENT STATEMENT: Informed consent was obtained from each participant or their surrogate decision makers. The study was approved by the UCSF Institutional Review Board.

KEY WORDS: network hyperexcitability; gamma oscillations; magnetoencephalography; phase amplitude coupling (PAC); Alzheimer's disease.

For Review Only

REFERENCES

1. Palop JJ, Chin J, Roberson ED*, et al*. Aberrant excitatory neuronal activity and compensatory remodeling of inhibitory hippocampal circuits in mouse models of Alzheimer's disease. *Neuron*. Sep 06 2007;55(5):697-711. doi:10.1016/j.neuron.2007.07.025

2. Busche MA, Chen X, Henning HA*, et al*. Critical role of soluble amyloid-beta for early hippocampal hyperactivity in a mouse model of Alzheimer's disease. *Proc Natl Acad Sci U S A*. May 29 2012;109(22):8740-5. doi:10.1073/pnas.1206171109

3. Busche MA, Hyman BT. Synergy between amyloid-beta and tau in Alzheimer's disease. *Nat Neurosci*. Oct 2020;23(10):1183-1193. doi:10.1038/s41593-020-0687-6

4. Vossel KA, Ranasinghe KG, Beagle AJ*, et al*. Incidence and impact of subclinical epileptiform activity in Alzheimer's disease. *Ann Neurol*. Dec 2016;80(6):858-870. doi:10.1002/ana.24794

5. Lam AD, Deck G, Goldman A, Eskandar EN, Noebels J, Cole AJ. Silent hippocampal seizures and spikes identified by foramen ovale electrodes in Alzheimer's disease. *Nat Med*. Jun 2017;23(6):678-680. doi:10.1038/nm.4330

6. Horvath AA, Papp A, Zsuffa J*, et al*. Subclinical epileptiform activity accelerates the progression of Alzheimer's disease: A long-term EEG study. *Clin Neurophysiol*. May 8 2021;doi:10.1016/j.clinph.2021.03.050

7. Wu JW, Hussaini SA, Bastille IM*, et al*. Neuronal activity enhances tau propagation and tau pathology in vivo. *Nat Neurosci*. Aug 2016;19(8):1085-92. doi:10.1038/nn.4328

8. Rodriguez GA, Barrett GM, Duff KE, Hussaini SA. Chemogenetic attenuation of neuronal activity in the entorhinal cortex reduces Abeta and tau pathology in the hippocampus. *PLoS Biol*. Aug 2020;18(8):e3000851. doi:10.1371/journal.pbio.3000851

For Review Only

9. Buzsaki G, Wang XJ. Mechanisms of gamma oscillations. Review. *Annual review of neuroscience*. 2012;35:203-25. doi:10.1146/annurev-neuro-062111-150444

10. Palop JJ, Mucke L. Network abnormalities and interneuron dysfunction in Alzheimer disease. *Nat Rev Neurosci*. Dec 2016;17(12):777-792. doi:10.1038/nrn.2016.141

11. Verret L, Mann EO, Hang GB*, et al*. Inhibitory interneuron deficit links altered network activity and cognitive dysfunction in Alzheimer model. In Vitro

Research Support, N.I.H., Extramural

Research Support, Non-U.S. Gov't. *Cell*. Apr 27 2012;149(3):708-21. doi:10.1016/j.cell.2012.02.046

12. Jia X, Kohn A. Gamma rhythms in the brain. *PLoS Biol*. Apr 2011;9(4):e1001045. doi:10.1371/journal.pbio.1001045

13. Tallon-Baudry C, Bertrand O. Oscillatory gamma activity in humans and its role in object representation. *Trends Cogn Sci*. Apr 1999;3(4):151-162. doi:10.1016/s1364-6613(99)01299-1

14. Fries P, Reynolds JH, Rorie AE, Desimone R. Modulation of oscillatory neuronal synchronization by selective visual attention. *Science*. Feb 23 2001;291(5508):1560-3. doi:10.1126/science.1055465

15. Jensen O, Colgin LL. Cross-frequency coupling between neuronal oscillations. *Trends in Cognitive Sciences*. Jul 2007;11(7):267-269. doi:10.1016/j.tics.2007.05.003

16. Siebenhuhner F, Wang SH, Arnulfo G*, et al*. Genuine cross-frequency coupling networks in human resting-state electrophysiological recordings. *PLoS Biol*. May 2020;18(5):e3000685. doi:10.1371/journal.pbio.3000685

For Review Only

17. van der Meij R, Kahana M, Maris E. Phase-amplitude coupling in human electrocorticography is spatially distributed and phase diverse. *J Neurosci*. Jan 4 2012;32(1):111-23. doi:10.1523/JNEUROSCI.4816-11.2012

18. Canolty RT, Edwards E, Dalal SS*, et al*. High gamma power is phase-locked to theta oscillations in human neocortex. Research Support, N.I.H., Extramural

Research Support, Non-U.S. Gov't

Research Support, U.S. Gov't, Non-P.H.S. *Science*. Sep 15 2006;313(5793):1626-8. doi:10.1126/science.1128115

19. Lakatos P, Shah AS, Knuth KH, Ulbert I, Karmos G, Schroeder CE. An oscillatory hierarchy controlling neuronal excitability and stimulus processing in the auditory cortex. Comparative Study. *Journal of neurophysiology*. Sep 2005;94(3):1904-11. doi:10.1152/jn.00263.2005

20. Fries P. A mechanism for cognitive dynamics: neuronal communication through neuronal coherence. Research Support, Non-U.S. Gov't

Review. *Trends in cognitive sciences*. Oct 2005;9(10):474-80. doi:10.1016/j.tics.2005.08.011

21. Ranasinghe KG, Kudo K, Hinkley L*, et al*. Neuronal synchrony abnormalities associated with subclinical epileptiform activity in early-onset Alzheimer's disease. *Brain*. Apr 18 2022;145(2):744-753. doi:10.1093/brain/awab442

22. Jack CR, Jr., Bennett DA, Blennow K*, et al*. NIA-AA Research Framework: Toward a biological definition of Alzheimer's disease. *Alzheimers Dement*. Apr 2018;14(4):535-562. doi:10.1016/j.jalz.2018.02.018

23. McKhann GM, Knopman DS, Chertkow H*, et al*. The diagnosis of dementia due to Alzheimer's disease: recommendations from the National Institute on Aging-Alzheimer's

For Review Only

Association workgroups on diagnostic guidelines for Alzheimer's disease. Consensus Development Conference, NIH

Research Support, Non-U.S. Gov't. *Alzheimers Dement*. May 2011;7(3):263-9. doi:10.1016/j.jalz.2011.03.005

24. Cai C, Kang H, Kirsch HE*, et al*. Comparison of DSSP and tSSS algorithms for removing artifacts from vagus nerve stimulators in magnetoencephalography data. *J Neural Eng*. Nov 12 2019;16(6):066045. doi:10.1088/1741-2552/ab4065

25. Dalal SS, Zumer JM, Guggisberg AG*, et al*. MEG/EEG source reconstruction, statistical evaluation, and visualization with NUTMEG. Research Support, N.I.H., Extramural

Research Support, Non-U.S. Gov't. *Comput Intell Neurosci*. 2011;2011:758973. doi:10.1155/2011/758973

26. Desikan RS, Segonne F, Fischl B*, et al*. An automated labeling system for subdividing the human cerebral cortex on MRI scans into gyral based regions of interest. *Neuroimage*. Jul 01 2006;31(3):968-80. doi:10.1016/j.neuroimage.2006.01.021

27. Pontecorvo MJ, Devous MD, Kennedy I*, et al*. A multicentre longitudinal study of flortaucipir (18F) in normal ageing, mild cognitive impairment and Alzheimer's disease dementia. *Brain*. Jun 1 2019;142(6):1723-1735. doi:10.1093/brain/awz090

28. Wang L, Benzinger TL, Su Y*, et al*. Evaluation of Tau Imaging in Staging Alzheimer Disease and Revealing Interactions Between beta-Amyloid and Tauopathy. *JAMA Neurol*. Sep 1 2016;73(9):1070-7. doi:10.1001/jamaneurol.2016.2078

29. Jagust WJ, Landau SM, Alzheimer's Disease Neuroimaging I. Temporal Dynamics of beta-Amyloid Accumulation in Aging and Alzheimer Disease. *Neurology*. Mar 2 2021;96(9):e1347-e1357. doi:10.1212/WNL.0000000000011524

For Review Only

30. de Flores R, Das SR, Xie L, *et al*. Medial Temporal Lobe Networks in Alzheimer's Disease: Structural and Molecular Vulnerabilities. *J Neurosci*. Mar 9 2022;42(10):2131-2141. doi:10.1523/JNEUROSCI.0949-21.2021

31. Chen X, Cassady KE, Adams JN, Harrison TM, Baker SL, Jagust WJ. Regional Tau Effects on Prospective Cognitive Change in Cognitively Normal Older Adults. *J Neurosci*. Jan 13 2021;41(2):366-375. doi:10.1523/JNEUROSCI.2111-20.2020

32. Fu HJ, Possenti A, Freer R, *et al*. A tau homeostasis signature is linked with the cellular and regional vulnerability of excitatory neurons to tau pathology. *Nat Neurosci*. Jan 2019;22(1):47-+. doi:10.1038/s41593-018-0298-7

33. Voglein J, Ricard I, Noachtar S, *et al*. Seizures in Alzheimer's disease are highly recurrent and associated with a poor disease course. *J Neurol*. Oct 2020;267(10):2941-2948. doi:10.1007/s00415-020-09937-7

34. Chang CW, Evans MD, Yu X, Yu GQ, Mucke L. Tau reduction affects excitatory and inhibitory neurons differently, reduces excitation/inhibition ratios, and counteracts network hypersynchrony. *Cell Rep*. Oct 19 2021;37(3):109855. doi:10.1016/j.celrep.2021.109855

35. Lopez-Pigozzi D, Laurent F, Brotons-Mas JR, *et al*. Altered Oscillatory Dynamics of CA1 Parvalbumin Basket Cells during Theta-Gamma Rhythmopathies of Temporal Lobe Epilepsy. *eNeuro*. Nov-Dec 2016;3(6)doi:10.1523/ENEURO.0284-16.2016

36. Sakkaki S, Barriere S, Bender AC, Scott RC, Lenck-Santini PP. Focal Dorsal Hippocampal Nav1.1 Knock Down Alters Place Cell Temporal Coordination and Spatial Behavior. *Cereb Cortex*. Jul 30 2020;30(9):5049-5066. doi:10.1093/cercor/bhaa101

37. Buzsaki G, Leung LW, Vanderwolf CH. Cellular bases of hippocampal EEG in the behaving rat. *Brain Res*. Oct 1983;287(2):139-71. doi:10.1016/0165-0173(83)90037-1

For Review Only

38. Fox SE, Wolfson S, Ranck JB, Jr. Hippocampal theta rhythm and the firing of neurons in walking and urethane anesthetized rats. *Exp Brain Res*. 1986;62(3):495-508. doi:10.1007/BF00236028

39. Pavlides C, Greenstein YJ, Grudman M, Winson J. Long-term potentiation in the dentate gyrus is induced preferentially on the positive phase of theta-rhythm. Research Support, Non-U.S. Gov't

Research Support, U.S. Gov't, Non-P.H.S.

Research Support, U.S. Gov't, P.H.S. *Brain Res*. Jan 26 1988;439(1-2):383-7.

40. Poe GR, Nitz DA, McNaughton BL, Barnes CA. Experience-dependent phase-reversal of hippocampal neuron firing during REM sleep. *Brain Res*. Feb 7 2000;855(1):176-80. doi:10.1016/s0006-8993(99)02310-0

41. Ranasinghe KG, Cha J, Iaccarino L*, et al*. Neurophysiological signatures in Alzheimer's disease are distinctly associated with TAU, amyloid-beta accumulation, and cognitive decline. *Sci Transl Med*. Mar 11 2020;12(534)doi:10.1126/scitranslmed.aaz4069

42. Babiloni C, Arakaki X, Azami H*, et al*. Measures of resting state EEG rhythms for clinical trials in Alzheimer's disease: Recommendations of an expert panel. *Alzheimers Dement*. Sep 2021;17(9):1528-1553. doi:10.1002/alz.12311

43. Iaccarino HF, Singer AC, Martorell AJ*, et al*. Gamma frequency entrainment attenuates amyloid load and modifies microglia. *Nature*. Dec 07 2016;540(7632):230-235. doi:10.1038/nature20587

44. Mably AJ, Gereke BJ, Jones DT, Colgin LL. Impairments in spatial representations and rhythmic coordination of place cells in the 3xTg mouse model of Alzheimer's disease. *Hippocampus*. Apr 2017;27(4):378-392. doi:10.1002/hipo.22697

For Review Only

45. Mondragon-Rodriguez S, Gu N, Manseau F, Williams S. Alzheimer's Transgenic Model Is Characterized by Very Early Brain Network Alterations and beta-CTF Fragment Accumulation: Reversal by beta-Secretase Inhibition. *Front Cell Neurosci*. May 8 2018;12doi:ARTN 121

10.3389/fncel.2018.00121

46. Stam CJ, van Cappellen van Walsum AM, Pijnenburg YA*, et al*. Generalized synchronization of MEG recordings in Alzheimer's Disease: evidence for involvement of the gamma band. *J Clin Neurophysiol*. Dec 2002;19(6):562-74. doi:10.1097/00004691-200212000-00010

47. Casula EP, Pellicciari MC, Bonni S*, et al*. Decreased Frontal Gamma Activity in Alzheimer Disease Patients. *Ann Neurol*. Sep 2022;92(3):464-475. doi:10.1002/ana.26444

48. Rossini PM, Del Percio C, Pasqualetti P*, et al*. Conversion from mild cognitive impairment to Alzheimer's disease is predicted by sources and coherence of brain electroencephalography rhythms. *Neuroscience*. Dec 2006;143(3):793-803. doi:10.1016/j.neuroscience.2006.08.049

49. van Deursen JA, Vuurman EF, Verhey FR, van Kranen-Mastenbroek VH, Riedel WJ. Increased EEG gamma band activity in Alzheimer's disease and mild cognitive impairment. *J Neural Transm (Vienna)*. Sep 2008;115(9):1301-11. doi:10.1007/s00702-008-0083-y

50. Ribary U, Ioannides AA, Singh KD*, et al*. Magnetic field tomography of coherent thalamocortical 40-Hz oscillations in humans. *Proc Natl Acad Sci U S A*. Dec 15 1991;88(24):11037-41. doi:10.1073/pnas.88.24.11037

For Review Only

51. Cuesta P, Ochoa-Urrea M, Funke M*, et al*. Gamma band functional connectivity reduction in patients with amnestic mild cognitive impairment and epileptiform activity. *Brain Commun*. 2022;4(2):fcac012. doi:10.1093/braincomms/fcac012

52. Buzsaki G. *Rhythms of the brain*. Oxford University press; 2011.

53. McLardy T. Hippocampal formation of brain as detector-coder of temporal patterns of information. *Perspectives in Biology and Medicine*. 1959;2(4):443-452.

54. Lisman JE, Idiart MA. Storage of 7±2 short-term memories in oscillatory subcycles. *Science*. 1995;267(5203):1512-1515.

55. Jensen O. Maintenance of multiple working memory items by temporal segmentation. *Neuroscience*. 2006;139(1):237-249.

56. Fries P. Rhythms for Cognition: Communication through Coherence. *Neuron*. Oct 7 2015;88(1):220-35. doi:10.1016/j.neuron.2015.09.034

57. Sacks DD, Schwenn PE, McLoughlin LT, Lagopoulos J, Hermens DF. Phase–amplitude coupling, mental health and cognition: implications for adolescence. *Front Hum Neurosci*. 2021;15:622313.

For Review Only

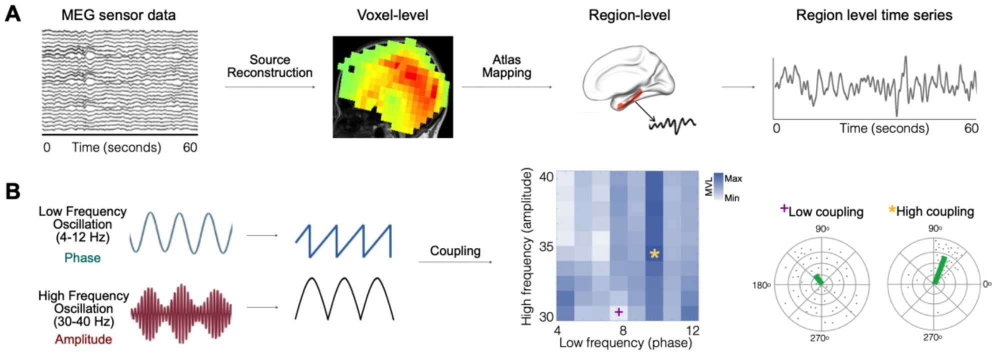

Figure 1: Estimation of gamma band PAC. MEG source-reconstructed region-level time series is mapped onto atlas parcellations to obtain time series for each cortical region of L and R entorhinal and L and R parahippocampal cortex. Each region-level time series was filtered at 4-12 Hz and 30-40 Hz to get phase and amplitude signals, respectively. Then, the coupling strength is measured using the mean vector length between the phase of 4-12 Hz signal and the amplitude of 30-40 Hz signal (B). Idealised depiction of high coupling and low coupling is shown in blue-to-white color gradient on the PAC grid. Polar plots corresponds to data points of complexed composite signals for the low and high PAC identified on the grid. Mean of these points (mean vector length) is represented in the green line. High coupling is represented by a large mean vector length and low coupling is represented by a small mean vector length. (Abbreviations: PAC, phase amplitude coupling)

215x78mm (300 x 300 DPI)

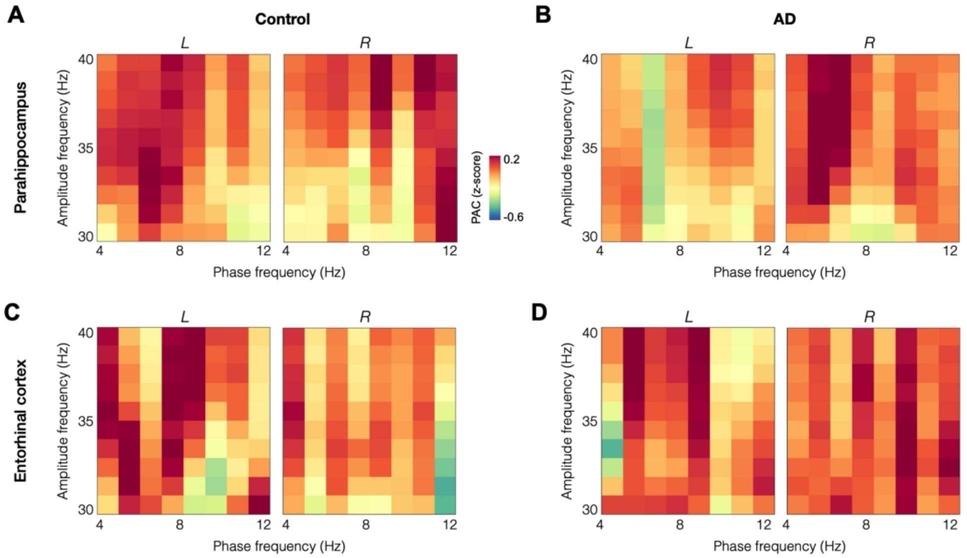

Figure 2: Gamma band PAC in patients with AD and age-matched controls in parahippocampal and entorhinal cortices. Gamma PAC within parahippocampal cortices, especially the left-parahippocampus showed reductions in patients with AD than controls (A-B). Gamma PAC within entorhinal cortices showed similar patterns in age-matched controls and in patients with AD and (C-D). (Abbreviations: AD, Alzheimer's disease; PAC, phase amplitude coupling)

199x118mm (300 x 300 DPI)

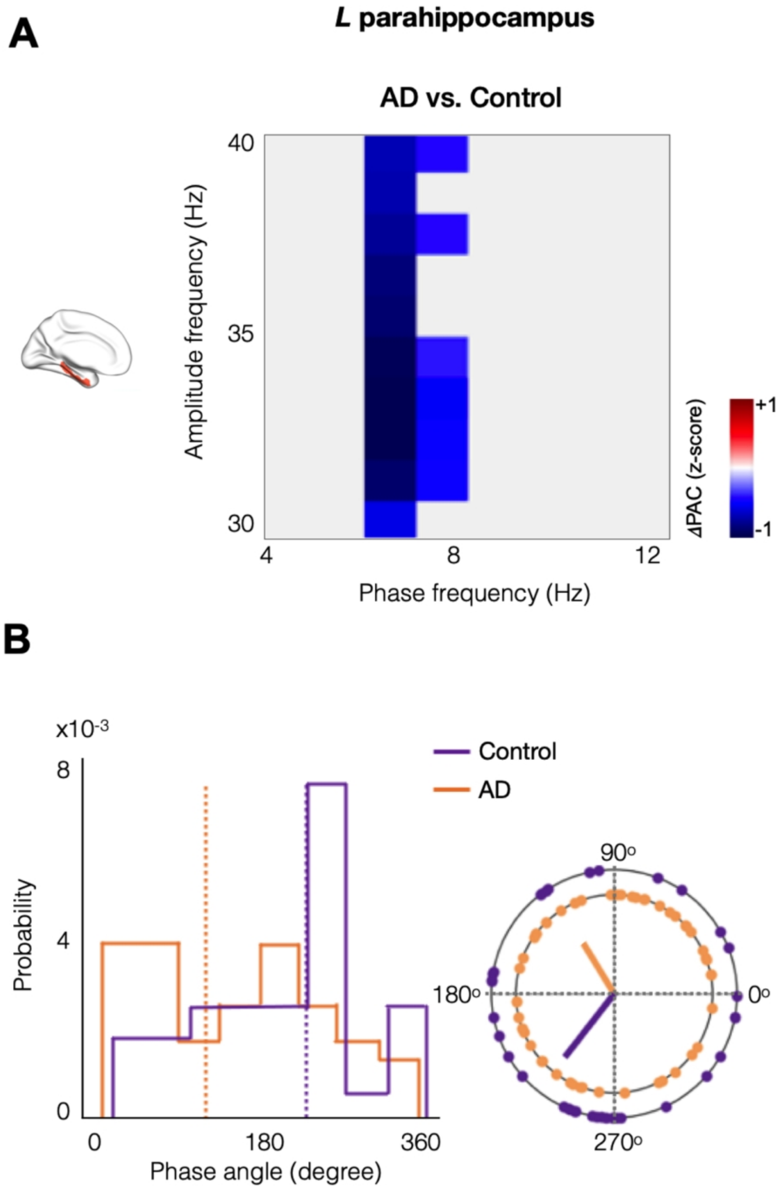

Figure 3: Patients with AD showed reduced gamma PAC within left parahippocampus. Patients with AD showed reduced coupling of gamma amplitude and theta (6-8 Hz) phase in the left parahippocampal cortex compared to age-matched controls (A). The blue-colored phase-amplitude coupling range showed statistically significant reductions in AD patients vs. age-matched controls in the left parahippocampus. The phase angle distribution in the left parahippocampal region, within the significant phase-amplitude coupling range (as indicated in blue in subplot A), showed that AD patients (orange dotted line; mean phase angle=121.7°) are leading age-matched controls (purple dotted line; mean phase angle=231.8°) (B). (Abbreviations: AD, Alzheimer's disease; PAC, phase amplitude coupling)

104x160mm (300 x 300 DPI)

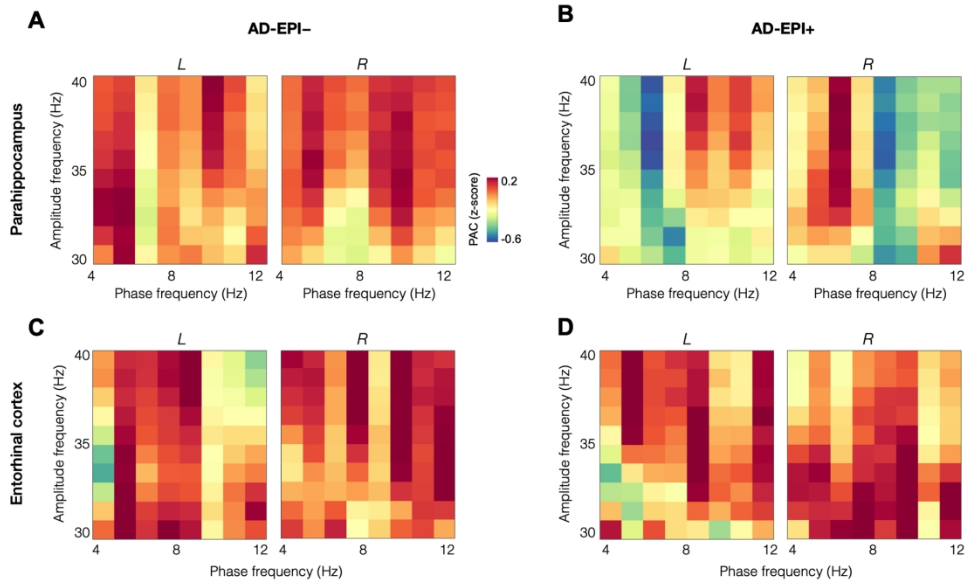

Figure 4: Gamma band PAC in AD-EPI− and AD-EPI+ in parahippocampal and entorhinal cortices. Gamma PAC within both left and right parahippocampal cortices, showed reductions in patients with AD-EPI+ compared to patients with AD-EPI− (A-B). Gamma PAC within entorhinal cortices showed similar patterns in AD-EPI− and AD-EPI+ (C-D). (Abbreviations: AD, Alzheimer's disease; PAC, phase amplitude coupling)

199x123mm (300 x 300 DPI)

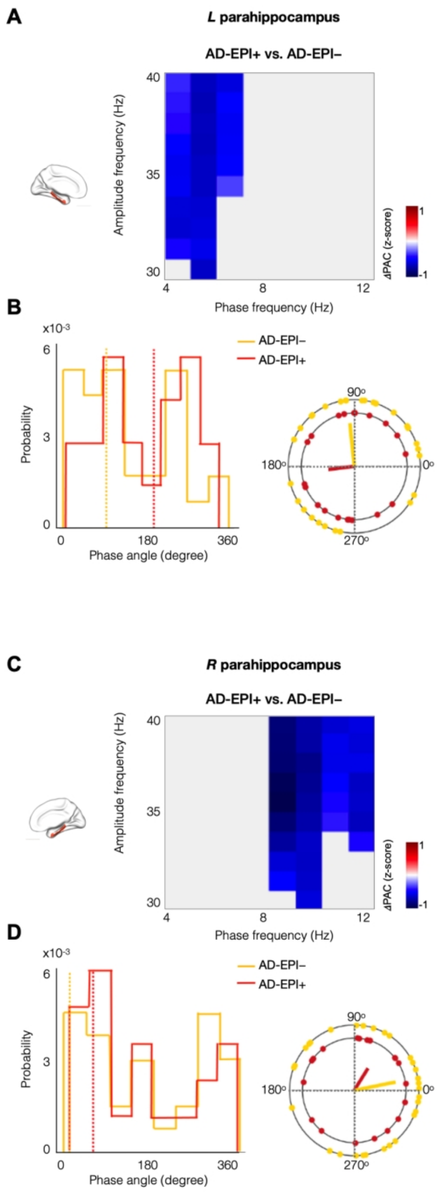

Figure 5: Patients with AD-EPI+ showed reduced gamma PAC in bilateral parahippocampal cortices compared to AD-EPI−. Patients with AD-EPI+ showed reduced theta-gamma PAC in the left parahippocampal region (A). The blue-colored phase-amplitude coupling range showed statistically reductions in AD-EPI+ patients vs. AD-EPI− patients in the left hippocampus (B). The phase angle distribution in the left parahippocampal region, within the significant phase-amplitude coupling range (as indicated in blue in subplot B), showed that AD-EPI− (yellow dotted line; mean phase angle=95.8°) are leading AD-EPI+ patients (red dotted line; mean phase angle=186.7°). Patients with AD-EPI+ showed reduced alpha-gamma PAC in the right parahippocampal cortex (C). The phase angle distribution in the right parahippocampal region, within the significant phase-amplitude coupling range (as indicated in blue in subplot C), showed that AD-EPI− (yellow dotted line; mean phase angle=11.5°) are leading AD-EPI+ patients (red dotted line; mean phase angle=57.8°; D). (Abbreviations: AD, Alzheimer's disease; AD-EPI+, AD patients with epileptiform activity; AD-EPI−, AD patients without epileptiform activity; PAC, phase amplitude coupling).

74x199mm (300 x 300 DPI)

SUPPLEMENT

1. Supplementary Tables

1. Supplementary Table 1: Participant Demographics and Clinical Characteristics

2. Supplementary Table 2: Neuropsychological Test Performance in patients with AD

3. Supplementary Table 3: Biomarkers of Alzheimer’s disease patients

For Review Only

1.1 Supplementary Table 1: Participant Demographics and Clinical Characteristics

Characteristic	Controls (N=35)	AD-EPI− (N=30)	AD-EPI+ (N=20)	*P* *
Age – yr	63.0 ± 5.8	60.7 ± 8.3	59.9 ± 6.7	0.122
Female sex – no. (%)	22 (62.8)	17 (56.7)	12 (60.0)	0.879
White – no. (%)[†]	25 (92.6)	27 (96.4)	19 (100.0)	0.449
Education – yr	17.4 ± 1.6	15.7 ± 2.6	17.0 ± 2.7	**0.012**[#]
Right handedness – no. (%)	28 (80.0)	25 (83.3)	18 (90.0)	0.629
Apo E ε4 carrier – no. (%)	4 (16.0)	12 (44.4)	9 (47.4)	**0.043**[#]
MMSE[‡]	29.6 ± 0.7	21.3 ± 5.8	21.5 ± 4.7	0.911
CDR[§]	0	1.0 (0.5 – 1.0)	1.0 (0.5 – 1.0)	0.351
CDR-SOB[§]	0	4.5 (3.5 – 5.0)	4.7 (3.3 – 6.5)	0.690
Age at disease onset	.	54.0 (50.0 -58.0)	53.5 (50.0 – 57.5)	0.882
Disease duration	.	5.4 (4.7 – 7.2)	4.8 (3.8 – 6.5)	0.440
Early onset AD – no. (%)	.	28 (93.3)	19 (95.0)	0.44
Atypical AD – no. (%)	.	11 (36.7)	7 (35.0)	0.904
Generalized slowing on LTM-EEG – no. (%)	.	7 (23.3)	6 (30.0)	0.598
Asymmetric / focal slowing on LTM-EEG – no. (%)	.	4 (13.3)	3 (15.0)	0.868
Generalized slowing on MEG-EEG – no. (%)	.	13 (43.3)	6 (30.0)	0.341
Asymmetric / focal slowing on MEG-EEG – no. (%)	.	5 (16.7)	8 (40.0)	0.065
On AChE-I – no. (%)	.	15 (50.0)	12 (60.0)	0.487
On memantine – no. (%)	.	1 (3.3)	1 (5.0)	0.768
On AChE-I & memantine – no. (%)	.	7 (23.3)	3 (15.0)	0.470
On antidepressants – no. (%)	.	15 (50.0)	11 (55.0)	0.729

Values for age, education, and MMSE are means ±SD.
Values for CDR, CDRSOB, age at disease onset and disease duration are medians with interquartile ranges in parentheses.
*Statistical tests: P values are reported from one-way-ANOVA with pairwise comparisons for Age and education, Pearson χ^2 test for sex, and Apolipoprotein-ε4 carrier, race and handedness, for the full cohort including the 3 groups. P values are reported Pearson χ^2 test between AD-EPI+ and AD-

For Review Only

EPI− for atypical AD, generalized slowing on EEG, asymmetric/focal closing on EEG, generalized slowing on MEG, asymmetric/focal closing on MEG, usage of AchE-I, memantine, combined AchEI and memantine theraly, and antidepressant use. P values for early onset AD, are reported from Fisher's exact test between AD-EPI+ and AD-EPI− . P values for the MMSE is reported from unpaired t-test and for CDR, CDRSOB, age at disease onset, and disease duration are reported from Wilcoxon-Mann-Whitney test, comparing the two patient cohorts.

†Race or ethnic group was self-reported. Two patients with AD and eight controls opted out from reporting the race.

# Pairwise comparison for education between controls and AD-EPI− showed statistically significant difference (P=0.011); pairwise analyses showed significant higher proportions of Apolipoprotein E ε4 carrier status in AD compared to controls (16% vs. 45% in controls and all-AD, P=0.0125), but no differences between the two AD cohorts (P=0.844).

‡Scores on the MMSE range from 0 to 30, with higher scores denoting better cognitive function.

§Scores on the CDR range from 0 to 3 and scores on the CDR-SOB range from 0 to 18, with higher scores denoting more disability.

Abbreviations: AChE-I, Acetylcholinesterase inhibitor; AD, Alzheimer's disease; AD-EPI−, AD patients without epileptiform activity; AD-EPI+, AD patients with epileptiform activity; ApoE, Apolipoprotein E; CDR, Clinical Dementia Rating; CDR-SOB, CDR-Sum of Boxes; EEG, electroencephalography; MEG, magnetoencephalography; MMSE, Mini Mental State Examination.

For Review Only

1.2 Supplementary table 2: Neuropsychological test performance in patients with AD*

Variable	AD-EPI−	AD-EPI+	*P*
Episodic memory function			
Visual free recall (Benson 10 minutes)	4.6 ± 4.2	4.6 ± 3.8	0.997
Short delay verbal memory (CVLT 30 seconds)	3.7 ± 2.3	3.6 ± 2.4	0.977
Verbal free recall (CVLT 10 minutes)	2.0 ± 2.3	2.2 ± 3.0	0.788
Executive function & working memory			
Design Fluency	5.5 ± 2.8	6.4 ± 4.7	0.462
Information processing speed (Stroop color naming)	43.9 ± 23.9	51.2 ± 22.0	0.405
Cognitive control (Stroop Inhibition)	19.6 ± 13.9	20.6 ± 17.4	0.853
Verbal working memory (Digit span forward)	5 (4 – 5)	5 (4 – 7)	0.934
Attention (Digit span backward)	3 (2 – 3)	3 (3 – 4)	0.114
Set shifting (Modified trails – speed)	0.2 ± 0.2	0.2 ± 0.2	0.755
Verbal learning (CVLT total score)	16.9 ± 6.1	17.0 ± 5.4	0.949
Language function			
Reading irregular words	6 (6 – 6)	6 (5 – 6)	0.065
Syntax comprehension	3.3 ± 1.4	3.3 ± 1.2	0.925
Verbal Agility	4.2 ± 1.6	4.1 ± 2.1	0.802
Boston Naming Test	13 (11 -14)	13 (10 – 14)	0.627
Lexical Fluency (D words/1 minute)	8.6 ± 4.6	10.9 ± 5.8	0.147
Category Fluency (Animals/1 minute)	11.5 ± 5.9	11.1 ± 5.5	0.789
Repetition	3.5 ± 1.3	3.4 ± 1.7	0.903
Visuospatial function			
Face discrimination (CATS – face matching)	11.1 ± 1.1	10.4 ± 1.4	0.091
Visuoconstruction (Benson copy)	10.4 ± 4.7	10.1 ± 5.2	0.873
Location discrimination (VOSP number location)	6.1 ± 2.6	5.9 ± 2.7	0.822
Calculations	2.6 ± 1.5	2.9 ± 1.3	0.585
Emotion naming (CATS – affect matching)	12.4 ± 1.6	11.0 ± 2.4	0.038

* Plus–minus values are means ±SD and remainder of values are medians with interquartile ranges in parentheses. MMSE=Mini-Mental State Examination; CDR=Clinical Dementia Rating; CDR-SOB=CDR Sum of Boxes; CVLT=California Verbal Learning Test containing 9 items; CATS=Comprehensive Affect Testing System; VOSP=Visual Object and Space Perception.

For Review Only

1.3 Supplementary table 3: Biomarkers of Alzheimer's disease patients

Patient number	Autopsy[‡]	CSF	Amyloid[§]	FDG[¶]
1	Confirmed	-	-	-
2	Confirmed	-	-	-
3	Confirmed	-	-	-
4	Confirmed	-	-	-
5	Confirmed	-	Positive	Positive
6	Confirmed	-	Positive	Positive
7	Confirmed	-	Positive	Positive
8	Confirmed	-	Positive	Positive
9	Confirmed	-	Positive	Positive
10	Confirmed	-	Positive	Positive
11	Confirmed	-	Positive	Positive
12	Confirmed	-	Positive	Positive
13	Confirmed	-	Positive	Positive
14	Confirmed	-	Positive	Positive
15	-	Aβ42=125.0 t-Tau=559.4 p-Tau= 82.0[†] Aβ42-Tau Index=0.14[†]		
16	-	Aβ42=240.3 t-Tau=1090.55 p-Tau=142.1[†] Aβ42-Tau Index=0.16[†]		
17	-	Aβ42=182.7 t-Tau=528.5 p-Tau=90.0[†] Aβ42-Tau Index=0.21[†]	Positive	Positive
18	-	Aβ42=294 t-Tau=833.65 p-Tau=113.55[†] Aβ42-Tau Index=0.24[†]	Positive	Positive

For Review Only

Patient number	Autopsy‡	CSF	Amyloid§	FDG¶
19	-	Aβ42=210.3<br>t-Tau=504.3 p-Tau=79.8†<br>Aβ42-Tau Index=0.25†		
20	-	Aβ42=399.5<br>t-Tau=527.6 p-Tau=70.5†<br>Aβ42-Tau Index=0.46†		
21	-	Aβ42=399.3<br>t-Tau=441.8 p-Tau=68.6†<br>Aβ42-Tau Index=0.52†		
22	-	Aβ42=473.7<br>t-Tau=366.6 p-Tau=74.75†<br>Aβ42-Tau Index=0.70†		
23	-	Aβ42=129¥<br>t-Tau=243¥<br>p-Tau=55¥	Positive	Positive
24	-	Aβ42=338.75<br>t-Tau=348.5 p-Tau=66.7†<br>Aβ42-Tau Index=0.52†	-	-
25	-	Aβ42=379.4<br>t-Tau=604.5 p-Tau=91.5†<br>Aβ42-Tau Index=0.4†	-	-
26	-	Abeta42 low, p-tau high. Interpretation: consistent with AD$	-	-
27	-	Abeta42 borderline low, p-tau high. Interpretation: consistent with AD$	-	-
28	-	-	Positive	Positive
29	-	-	Positive	Positive
30	-	-	Positive	Positive
31	-	-	Positive	Positive
32	-	-	Positive	Positive
33	-	-	Positive	Positive
34	-	-	Positive	Positive
35	-	-	Positive	Positive
36	-	-	Positive	Positive
37	-	-	Positive	Positive

For Review Only

Patient number	Autopsy‡	CSF	Amyloid§	FDG¶
38	-	-	Positive	Positive
39	-	-	Positive	Positive
40	-	-	Positive	Positive
41	-	-	Positive	Positive
42	-	-	Positive	Positive
43	-	-	Positive	Positive
44	-	-	Positive	Positive
45	-	-	Positive	Positive
46	-	-	Positive	Positive
47	-	-	Positive	Positive
48	-	-	Positive	Positive
49	-	-	Positive	Positive
50	-	-	Positive	Positive

For Review Only

Abbreviations: Aβ42 = amyloid-β peptide ending in amino acid residue 42; CSF = cerebrospinal fluid; L = left; MRI = magnetic resonance imaging; p-Tau = tau phosphorylated at threonine 181; R = right; t-Tau = total tau.

‡ Alzheimer's disease was confirmed by autopsy according to National Institute on Aging–Reagan Institute criteria.

†Values supporting a diagnosis of Alzheimer's disease are p-Tau level >61 pg/ml and Aβ42-Tau Index <1.0 (Athena Diagnostics).

¥ Values supporting a diagnosis of Alzheimer's disease are Aβ42 level <192 pg/ml, t-Tau level >93 pg/ml, and p-Tau level >23 pg/ml (Alzheimer's Disease Neuroimaging Initiative Biomarker Core at the University of Pennsylvania).

$ Values supporting a diagnosis of Alzheimer’s disease are indicated in the comments. (Alzheimer's Disease Neuroimaging Initiative Biomarker Core at the University of San Diego).

§ Positron emission tomography agent was ^{18}F-AV-45 for patients 17, 18, 33, 34, 36, 37, 40 and 41, and ^{11}C-Pittsburgh compound B for the remainder of the patients.

¶ Positron emission tomography imaging with ^{18}F-fluorodeoxyglucose (^{18}F-FDG) showed patterns of hypometabolism consistent with Alzheimer’s disease.

For Review Only
