# Supplementary material for: Abnormal gamma phase-amplitude coupling in the parahippocampal cortex is associated with network hyperexcitability in Alzheimer’s disease
Source: Brain Commun. 2024 Apr 9;6(2):fcae121. doi: 10.1093/braincomms/fcae121 (PMC11043655; doi:10.1093/braincomms/fcae121)
Supplement: fcae121_Supplementary_Data [file fcae121_supplementary_data.zip › Supplementary_material.docx]

**SUPPLEMENT**

**Supplementary Tables**

Supplementary Table 1: Participant Demographics and Clinical Characteristics

Supplementary Table 2: Neuropsychological Test Performance in patients with AD

Supplementary Table 3: Biomarkers of Alzheimer’s disease patients

**Codes**

Matlab script to generate PAC values and peak angles.

Python script for Permutation Cluster test between AD and Controls, and AD-EPI+ vs. AD-EPI-.

**Supplementary Table 1: Participant Demographics and Clinical Characteristics**

| **Characteristic** | **Controls**  **(N=35)** | **AD-EPI−**  **(N=30)** | **AD-EPI+**  **(N=20)** | ***P* *** |
| --- | --- | --- | --- | --- |
| Age – yr | 63.0 ± 5.8 | 60.7 ± 8.3 | 59.9 ± 6.7 | 0.122 |
| Female sex – no. (%) | 22 (62.8) | 17 (56.7) | 12 (60.0) | 0.879 |
| White – no. (%)^†^ | 25 (92.6) | 27 (96.4) | 19 (100.0) | 0.449 |
| Education – yr | 17.4 ± 1.6 | 15.7 ± 2.6 | 17.0 ± 2.7 | **0.012**^#^ |
| Right handedness – no. (%) | 28 (80.0) | 25 (83.3) | 18 (90.0) | 0.629 |
| Apo E ɛ4 carrier – no. (%) | 4 (16.0) | 12 (44.4) | 9 (47.4) | **0.043**^#^ |
| MMSE^‡^ | 29.6 ± 0.7 | 21.3 ± 5.8 | 21.5 ± 4.7 | 0.911 |
| CDR^§^ | 0 | 1.0 (0.5 – 1.0) | 1.0 (0.5 – 1.0) | 0.351 |
| CDR-SOB^§^ | 0 | 4.5 (3.5 – 5.0) | 4.7 (3.3 – 6.5) | 0.690 |
| Age at disease onset | . | 54.0 (50.0 -58.0) | 53.5 (50.0 – 57.5) | 0.882 |
| Disease duration | . | 5.4 (4.7 – 7.2) | 4.8 (3.8 – 6.5) | 0.440 |
| Early onset AD – no. (%) | . | 28 (93.3) | 19 (95.0) | 0.44 |
| Atypical AD – no. (%) | . | 11 (36.7) | 7 (35.0) | 0.904 |
| Generalized slowing on LTM-EEG – no. (%) | . | 7 (23.3) | 6 (30.0) | 0.598 |
| Asymmetric / focal slowing on LTM-EEG – no. (%) | . | 4 (13.3) | 3 (15.0) | 0.868 |
| Generalized slowing on MEG-EEG – no. (%) | . | 13 (43.3) | 6 (30.0) | 0.341 |
| Asymmetric / focal slowing on MEG-EEG – no. (%) | . | 5 (16.7) | 8 (40.0) | 0.065 |
| On AChE-I – no. (%) | . | 15 (50.0) | 12 (60.0) | 0.487 |
| On memantine – no. (%) | . | 1 (3.3) | 1 (5.0) | 0.768 |
| On AChE-I & memantine – no. (%) | . | 7 (23.3) | 3 (15.0) | 0.470 |
| On antidepressants – no. (%) | . | 15 (50.0) | 11 (55.0) | 0.729 |

Values for age, education, and MMSE are means ±SD.

Values for CDR, CDRSOB, age at disease onset and disease duration are medians with interquartile ranges in parentheses.

*Statistical tests: P values are reported from one-way-ANOVA with pairwise comparisons for Age and education, Pearson 𝜒^2^ test for sex, and Apolipoprotein-ɛ4 carrier, race and handedness, for the full cohort including the 3 groups. P values are reported Pearson 𝜒^2^ test between AD-EPI+ and AD-EPI− for atypical AD, generalized slowing on EEG, asymmetric/focal closing on EEG, generalized slowing on MEG, asymmetric/focal closing on MEG, usage of AchE-I, memantine, combined AchEI and memantine theraly, and antidepressant use. P values for early onset AD, are reported from Fisher’s exact test between AD-EPI+ and AD-EPI− . P values for the MMSE is reported from unpaired t-test and for CDR, CDRSOB, age at disease onset, and disease duration are reported from Wilcoxon-Mann-Whitney test, comparing the two patient cohorts.

†Race or ethnic group was self-reported. Two patients with AD and eight controls opted out from reporting the race.

^#^ Pairwise comparison for education between controls and AD-EPI− showed statistically significant difference (P=0.011); pairwise analyses showed significant higher proportions of Apolipoprotein E ɛ4 carrier status in AD compared to controls (16% vs. 45% in controls and all-AD, P=0.0125), but no differences between the two AD cohorts (P=0.844).

^‡^Scores on the MMSE range from 0 to 30, with higher scores denoting better cognitive function.

^§^Scores on the CDR range from 0 to 3 and scores on the CDR-SOB range from 0 to 18, with higher scores denoting more disability.

Abbreviations: AChE-I, Acetylcholinesterase inhibitor; AD, Alzheimer’s disease; AD-EPI−, AD patients without epileptiform activity; AD-EPI+, AD patients with epileptiform activity; ApoE, Apolipoprotein E; CDR, Clinical Dementia Rating; CDR-SOB, CDR-Sum of Boxes; EEG, electroencephalography; MEG, magnetoencephalography; MMSE, Mini Mental State Examination.

**Supplementary table 2: Neuropsychological test performance in patients with AD***

| **Variable** | **AD-EPI−** | **AD-EPI+** | ***P*** |
| --- | --- | --- | --- |
| ***Episodic memory function*** |  |  |  |
| Visual free recall (Benson 10 minutes) | 4.6 ± 4.2 | 4.6 ± 3.8 | 0.997 |
| Short delay verbal memory (CVLT 30 seconds) | 3.7 ± 2.3 | 3.6 ± 2.4 | 0.977 |
| Verbal free recall (CVLT 10 minutes) | 2.0 ± 2.3 | 2.2 ± 3.0 | 0.788 |
| ***Executive function & working memory*** |  |  |  |
| Design Fluency | 5.5 ± 2.8 | 6.4 ± 4.7 | 0.462 |
| Information processing speed (Stroop color naming) | 43.9 ± 23.9 | 51.2 ± 22.0 | 0.405 |
| Cognitive control (Stroop Inhibition) | 19.6 ± 13.9 | 20.6 ± 17.4 | 0.853 |
| Verbal working memory (Digit span forward) | 5 (4 – 5) | 5 ( 4 – 7) | 0.934 |
| Attention (Digit span backward) | 3 (2 – 3) | 3 (3 – 4) | 0.114 |
| Set shifting (Modified trails – speed) | 0.2 ± 0.2 | 0.2 ± 0.2 | 0.755 |
| Verbal learning (CVLT total score) | 16.9 ± 6.1 | 17.0 ± 5.4 | 0.949 |
| ***Language function*** |  |  |  |
| Reading irregular words | 6 (6 – 6) | 6 (5 – 6) | 0.065 |
| Syntax comprehension | 3.3 ± 1.4 | 3.3 ± 1.2 | 0.925 |
| Verbal Agility | 4.2 ± 1.6 | 4.1 ± 2.1 | 0.802 |
| Boston Naming Test | 13 (11 -14) | 13 (10 – 14) | 0.627 |
| Lexical Fluency (D words/1 minute) | 8.6 ± 4.6 | 10.9 ± 5.8 | 0.147 |
| Category Fluency (Animals/1 minute) | 11.5 ± 5.9 | 11.1 ± 5.5 | 0.789 |
| Repetition | 3.5 ± 1.3 | 3.4 ± 1.7 | 0.903 |
| ***Visuospatial function*** |  |  |  |
| Face discrimination (CATS – face matching) | 11.1 ± 1.1 | 10.4 ± 1.4 | 0.091 |
| Visuoconstruction (Benson copy) | 10.4 ± 4.7 | 10.1 ± 5.2 | 0.873 |
| Location discrimination (VOSP number location) | 6.1 ± 2.6 | 5.9 ± 2.7 | 0.822 |
| ***Calculations*** | 2.6 ± 1.5 | 2.9 ± 1.3 | 0.585 |
| ***Emotion naming*** (CATS – affect matching) | 12.4 ± 1.6 | 11.0 ± 2.4 | 0.038 |

* Plus–minus values are means ±SD and remainder of values are medians with interquartile ranges in parentheses. MMSE=Mini-Mental State Examination; CDR=Clinical Dementia Rating; CDR-SOB=CDR Sum of Boxes; CVLT=California Verbal Learning Test containing 9 items; CATS=Comprehensive Affect Testing System; VOSP=Visual Object and Space Perception.

**Supplementary table 3: Biomarkers of Alzheimer’s disease patients**

| **Patient number** | **Autopsy**^‡^ | **CSF** | **Amyloid**^§^ | **FDG**^¶^ |
| --- | --- | --- | --- | --- |
| 1 to 4 | Confirmed | - | - | - |
| 5 to 14 | Confirmed | - | Positive | Positive |
| 15 | - | Aβ_42_=125.0, t-Tau=559.4 p-Tau= 82.0^†^, Aβ_42_-Tau Index=0.14^†^ |  |  |
| 16 | - | Aβ_42_=240.3, t-Tau=1090.55 p-Tau=142.1^†^, Aβ_42_-Tau Index=0.16^†^ |  |  |
| 17 | - | Aβ_42_=182.7, t-Tau=528.5 p-Tau=90.0^†^, Aβ_42_-Tau Index=0.21^†^ | Positive | Positive |
| 18 | - | Aβ_42_=29, t-Tau=833.65 p-Tau=113.55^†^, Aβ_42_-Tau Index=0.24^†^ | Positive | Positive |
| 19 | - | Aβ_42_=210.3, t-Tau=504.3 p-Tau=79.8^†^, Aβ_42_-Tau Index=0.25^†^ |  |  |
| 20 | - | Aβ_42_=399.5, t-Tau=527.6 p-Tau=70.5^†^, Aβ_42_-Tau Index=0.46^†^ |  |  |
| 21 | - | Aβ_42_=399.3, t-Tau=441.8 p-Tau=68.6^†^, Aβ_42_-Tau Index=0.52^†^ |  |  |
| 22 | - | Aβ_42_=473.7, t-Tau=366.6 p-Tau=74.75^†^, Aβ_42_-Tau Index=0.70^†^ |  |  |
| 23 | - | Aβ_42_=129^¥^, t-Tau=243^¥^, p-Tau=55^¥^ | Positive | Positive |
| 24 | - | Aβ_42_=338.75, t-Tau=348.5 p-Tau=66.7^†^, Aβ_42_-Tau Index=0.52^†^ | - | - |
| 25 | - | Aβ_42_=379.4, t-Tau=604.5 p-Tau=91.5^†^, Aβ_42_-Tau Index=0.4^†^ | - | - |
| 26 | - | Aβ_42_ low, p-tau high, Interpretation: consistent with AD^$^ | - | - |
| 27 | - | Aβ_42_ borderline low, p-tau high, consistent with AD^$^ | - | - |
| 28 to 50 | - | - | Positive | Positive |

Abbreviations: Aβ42 = amyloid-β peptide ending in amino acid residue 42; CSF = cerebrospinal fluid; L = left; MRI = magnetic resonance imaging; p-Tau = tau phosphorylated at threonine 181; R = right; t-Tau = total tau.

‡ Alzheimer’s disease was confirmed by autopsy according to National Institute on Aging–Reagan Institute criteria.

†Values supporting a diagnosis of Alzheimer’s disease are p-Tau level >61 pg/ml and Aβ42-Tau Index <1.0 (Athena Diagnostics).

¥ Values supporting a diagnosis of Alzheimer’s disease are Aβ42 level <192 pg/ml, t-Tau level >93 pg/ml, and p-Tau level >23 pg/ml (Alzheimer's Disease Neuroimaging Initiative Biomarker Core at the University of Pennsylvania).

$ Values supporting a diagnosis of Alzheimer’s disease are indicated in the comments. (Alzheimer's Disease Neuroimaging Initiative Biomarker Core at the University of San Diego).

§ Positron emission tomography agent was ^18^F-AV-45 for patients 17, 18, 33, 34, 36, 37, 40 and 41, and ^11^C-Pittsburgh compound B for the remainder of the patients.

¶ Positron emission tomography imaging with ^18^F-fluorodeoxyglucose (^18^F-FDG) showed patterns of hypometabolism consistent with Alzheimer’s disease.

**Codes**

- 1. **Matlab script to generate PAC values and peak angles.**

%% Keep PACTools by EEGLab in the same path

%% [0] prep

clear all;

close all;

%% load time course data

load 'DK_timecourse_10.mat'

nROIAll=size(DK_timecourse,1);%68

nTimeAll=size(DK_timecourse,2);%600Hz*60sec=36000

srate=600;

%% set paramters (change values as you like)

ROIs=1:nROIAll; % must be a subset of 1:nROIAll

nROI=numel(ROIs);

nTime=nTimeAll; % must be smaller than or equal to nTimeAll

dataname='demo'; % name of output. i.e., <dataname>.set and <dataname>.mat

lowFreqRange=[4 12];

nLowFreq=1;

highFreqRange=[30 40];

nHighFreq=1;

%% setup data array

RT_data=zeros(nROI,nTime);

for iROI=1:nROI

RT_data(iROI,1:nTime)=DK_timecourse(ROIs(iROI),1:nTime);

end

%% save timecourse data as "<dataname>.set"

save_as_set(RT_data,dataname,srate);

data = pop_loadset('filename',[dataname '.set']);

%% compute PAC

%methodLst = {'mvlmi','klmi','glm','plv','instmipac'};

methodLst = {'mvlmi'};

for imethod=1:numel(methodLst)

method=methodLst{imethod};

data = pop_pac(data,'Channels',lowFreqRange,highFreqRange,...

1:nROI,1:nROI,'method',method,'nboot',200,'alpha',0.05,...

'nfreqs1',nLowFreq,'nfreqs2',nHighFreq,'freqscale','linear',...

'bonfcorr',1);

end

%% save

save([dataname '.mat'],'data')

1. **Python script for Permutation Cluster test between AD and Controls, and AD-EPI+ and AD-EPI-**

import numpy as np

from scipy import stats as stats

import mne

from mne.stats import permutation_cluster_test

#_______________________________________________________________

# load the data that has PAC values for AD and Controls (nSubjects x nROI x nAmpbins x nPhasebins)

data_spike20=spike20 #AD-Epi+

data_nospike30=nospike30 #AD-Epi-

data_epi=np.concatenate((data_spike20,data_nospike30),axis=0) #AD

data_cont=cont #Control

nROI=cont.shape[1] # 68 regions from DK atlas

#_________________________________________________

conds='AD_Cont' #'AD_Cont' or 'spike_nospike'

#_________________________________________________

# independent t-test for clusters

def stat_fun(*args):

return mne.stats.ttest_ind_no_p(args[0],args[1], equal_var=True, sigma=0.0)

#____________________________________________________

if conds=='AD_Cont':

print('AC')

##for AD vs. Control

cond1=data_epi

cond2=data_cont

p_threshold=0.05

n_subjects1=cond1.shape[0]

n_subjects2=cond2.shape[0]

sig_region=[]

sig_cluster=[]

sig_T=[]

t_threshold=stats.distributions.f.ppf(1. - p_threshold / 2.,

n_subjects1 - 1, n_subjects2 - 1)

for r in range(0,nROI):

# perform permutation cluster test

T,cluster,cluster_pvalues,H0=permutation_cluster_test([cond1[:,r,:,:],cond2[:,r,:,:]],n_permutations=1024,threshold=t_threshold,out_type='mask',tail=0,seed=np.random.seed(0),stat_fun=stat_fun)

# pick clusters p<=0.05

for c, p_val in zip(cluster, cluster_pvalues):

if p_val <= 0.05:

sig_region.append(r)

sig_cluster.append(c)

sig_T.append(T)

elif conds=='spike_nospike':

print('epi')

#for Epi+ vs Epi-

cond3=data_spike20

cond4=data_nospike30

p_threshold=0.05

n_subjects3=cond3.shape[0]

n_subjects4=cond4.shape[0]

sig_region=[]

sig_cluster=[]

sig_T=[]

t_threshold=stats.distributions.f.ppf(1. - p_threshold / 2.,

n_subjects3 - 1, n_subjects4 - 1)

for r in range(0,nROI):

T,cluster,cluster_pvalues,H0=permutation_cluster_test([cond3[:,r,:,:],cond4[:,r,:,:]],n_permutations=1024,threshold=t_threshold,out_type='mask',tail=0,seed=np.random.seed(0),stat_fun=stat_fun)

for c, p_val in zip(cluster, cluster_pvalues):

if p_val <= 0.05:

sig_region.append(r)

sig_cluster.append(c)

sig_T.append(T)
