# Supplementary material for: Abnormal gamma phase-amplitude coupling in the parahippocampal cortex is associated with network hyperexcitability in Alzheimer’s disease
Source: Brain Commun. 2024 Apr 9;6(2):fcae121. doi: 10.1093/braincomms/fcae121 (PMC11043655; doi:10.1093/braincomms/fcae121)
Supplement: fcae121_Supplementary_Data [file Original Submission - Author's Response.pdf › Original Submission - Author's Response.pdf]

Response to Reviewers' Comments

We thank the referees for reviewing our manuscript and providing valuable feedback. We are extremely pleased that reviewers found several strengths and laudable aspects in our study and made several important and helpful suggestions to improve the manuscript. We have incorporated all the suggestions in this revision. What follows is a detailed point-by-point response to all the points raised by each reviewer. The revisions are marked in yellow-highlight in the revised manuscript. The page-numbers and paragraph numbers mentioned at each response below refer to the PDF document of the revised manuscript which also indicates the revisions in *yellow highlight.*

Reviewer #1

This is a very interesting manuscript where authors use Phase-amplitude coupling (PAC) to access local gamma activity in the medial temporal lobe as a metric of neuronal excitability. Their results using a well-designed human cohort are compelling and indicate specific patterns associated with network hyperexcitability. This information can be very helpful to further examine hyperexcitability across different stages of pathology and its potential use as biomarker. I have only minor comments.

1. **In p11, Ln 20, it is not very clear whether the authors are referring to Primary age-related tauopathy. A more clear explanation in the text will help with clarity for the reader.**

Thank you for the comment. We have now explicitly clarified our statement using the term ‘primary age related tauopathy’ in the revised manuscript. *(page: 12, lines: 13-14)*

2. **Please discuss why the left parahippocampal regions were more affected in AD**

We completely agree with the reviewer’s observation that, in our study, patients with AD showed reduced gamma PAC in the left parahippocampus but not in the right parahippocampus, when compared to controls. In general, the typical late-onset AD, which is the most common clinical presentation, does not show hemispheric lateralization in cortical atrophy as well as regional tau accumulations. However, in the atypical presentations of AD, especially in the logopenic variant pf primary progressive aphasia as well as the early onset AD with predominant dysexecutive symptoms, left hemisphere is affected more than the right hemisphere in both neuronal loss as well as tau accumulations. As our cohort consists of ~90% of early onset AD and ~10% of atypical AD presentations as shown in the Supplementary table -1, this is a likely reason which makes the left hemisphere more susceptible compared to the right hemisphere in this cohort. We have now included this point in our revised discussion. *(page: 15, lines: 22-25; page: 16, lines: 1-6;)*

3. **The manuscript appropriately use AD-EPI- and AD-EPI+ phenotypes to facilitate comparisons, but more likely than a binomial distribution, hyperexcitability is found as a continuum in the clinical settings where AD-EPI+ is at one end of this continuum. Indeed, proexcitatory synaptic imbalance in AD correlate with worse cognitive performance and more neuropathology across the continuum of AD (PMID: 36538112), please discuss the utility of PAC to explore this continuum and other brain regions.**

We completely agree with the reviewer that hyperexcitability is a continuum in AD rather than a separate phenotypic entity and in our revised manuscript we discuss how PAC can be a useful quantitative metric in clinical populations. *(page: 17, lines: 3-17)*

Reviewer #2

Phase Amplitude Coupling (PAC) and its concordance with magnetoencephalography (MEG) and electroencephalography (EEG) associated characterization of a statistically well-powered group of AD subjects that can be divided into ones showing epileptic activity and with a control cohort has been attempted here to understand whether these parameters can be therapeutically translated to the clinic for diagnosing AD. Here are the major and minor points that may be important to consider for the current manuscript:

1. **Page 6 - Line 1-8 - While the scientific premise of using PAC is innovative in this approach, it is not very commonplace for the journal readers to be able to appreciate the gravity of the approach as presented here. The authors may consider a sentence or two in the introduction that clearly highlights how PAC goes beyond earlier EEG studies both for future approaches as well as the possibility to mine previously obtained data.**

Thank you for this suggestion. We now mention the importance of PAC as a quantitative metric in our introduction. Please also see that, in response to the 3rd question from Reviewer #1, we have also included a separate paragraph in the discussion section addressing this point. *(Introduction, page 5, lines: 7-10; Discussion, page: 17, lines: 3-17)*

2. **Page 6 – Line 11-12 – The audience would benefit by the understanding of why the coupling of the specified frequencies are more beneficial than the conventionally used ones (if that is done). Moreover, it would also be very relevant for the authors to demonstrate how this range is ideal for the PAC analysis being considered here by either using previous references or by providing preliminary data that was done in earlier studies that refined the outcome thus allowing the authors to explore this premise.**

Thank you for the suggestion. We have now included additional details into the methods section of the revised manuscript explaining the relevance of the specific frequency bands in relation to AD pathophysiology and also citing previous studies. *(page: 7, line: 24; page 8, lines 1-9)*

3. **Page 5 – Line 13-15 – This statement, in the opinion of this reviewer, seems to demonstrate a missed opportunity where the excitability alone was considered in the analysis. It is perhaps important for the authors to lay the rationale for why there was no focus on looking at signals that were statistically elevated in control subjects but missing in the AD patients which would have explained how the entorhinal PAC did not show any aberrant activity while being the foremost affected in AD patients.**

Thank you for the comment. We now clarify that we indeed compare patients with AD and age-matched controls (as shown in Figure 2 of the paper). We have revised the introduction of the manuscript explaining the details of specific cohort comparisons in our study which includes both AD vs. control and AD-EPI+ vs. AD-EPI- *(page: 5, lines: 14-18)*

4. **Page 5 – Line 21-24 – The age group of subjects selected for the study is fairly early stages in the clinical progression of the disease – can the authors elaborate the practical or the scientific premise for why different age groups were not considered to allow the readers to appreciate the importance of the age range mentioned here.**

Thank you for the comment. We agree with the reviewer that our cohort represents a fairly early stage of presentations and also overrepresents the early onset AD presentations. This is contributed by the current clinical cohort which was collected in part as an observational study to examine the incidence of subclinical epileptiform activity in AD patents. As epileptic manifestations

in AD were predicted as early events in the clinical course of the disease and was more prevalent in early onset AD, the original study was specifically geared to collect data from a sample which richly represented these demographic features. Given the extremely demand on the resources to identify subclinical epileptic events in electrophysiological recordings by expert neurophysiologists and epileptologists, which we have set as the standard to categorically identify patients as AD-EPI+ and AD-EPI-, we have relied on the current cohort as a convenience sample. As we have acknowledged under the study limitations, the current sample does indeed limits our ability to generalize the findings to larger AD populations. Notwithstanding such constraints, as our sample robustly represents the AD-EPI+ phenotype it facilitates the development of quantitative metrics of hyperexcitability such as reduced gamma PAC, which otherwise maybe missed in a cohort with a smaller fraction of AD-EPI+ representation. We have now further elaborated this point in our revised manuscript. *(page: 17, lines: 23-25; page: 18, lines: 1-5)*

5. **Page 7 – Line 1-9 – The authors need to provide the distribution of male and female subjects in these studies at this level also in addition to the results section, since there is a strong disproportional representation of AD in females. This also raises the questions as to whether the authors need to consider sex/gender as a variable in interpreting the outcomes of the current study.**

We thank the reviewer for this important comment. We have now included the distribution of male and female percentages into the cohort descriptions *(page: 10, lines: 6-8*. In addition, these data are also included in the Supplementary table-1. We agree that investigating sex as a biological modifier is an important step in analyses like ours. However, our current sample sized are underpowered to detect specific sex related effects, and we acknowledge in our 'limitations' section that these should be addressed in future studies with larger cohorts *(page: 18, lines: 5-8)*. Following the reviewers suggestion a post-hoc analysis using a GLM to examine the group-by-sex interaction for the significant group differences of PAC which was reported in our cohort. This analysis showed no significant interaction with sex in AD vs. controls group comparison (F=3.01, P=0.098; 95% confidence intervals for females and males, respectively: AD, -0.47 - 0.55; control, -1.12 - 0.13), as well as in the AD-EPI+ vs. AD-EPI- group comparisons (Left parahippocampus: F=0.06, P=0.805; 95% confidence intervals for females and males, respectively: AD-EPI+, -0.68 - 0.65; AD-EPI-, -0.63 - 0.44; Right parahippocampus: F=0.07, P=0.791; 95% confidence intervals for females and males, respectively: AD-EPI+, -0.73 - 1.08; AD-EPI-, -0.67 - 0.79)

6. **Page 7 – 11-25 – Can the authors elaborate on whether the studies on different patients were conducted at the same time of the day? There is a definitive component of circadian rhythm disruptions that needs to be considered for studying the progression of dementia. This necessitates that the authors, at the very least, consider reporting the time of the day and the state of the subjects (awareness/sleepiness) that the data was collected. Perhaps, if the statistical power remains intact, pooling the studies done at the same time of day with consideration to sex/gender may provide insights in PAC that may be more relevant from a clinical point of view.**

Thank you for this important point. Yes, the PAC analysis was done using a 60 second, awake, resting data epoch from the MEG/EEG data recording and the MEG data were collected from all participants between 9.30 - 11.00 AM using a uniform data collection protocol. Specifically, our study protocol included the participants to be admitted to the hospital the previous night for overnight long-term EEG with video monitoring, followed by an hour long MEG/EEG recording while at rest with eyes closed, on the next day morning between 9.30 - 11.00 AM. We have now included these details into the methods section of the revised manuscript. *(page: 6; lines: 16-20)*

7. **Page 13 – Line 1-5 – The discussion by the authors to highlight the importance of neuronal excitability in the progression of AD-tauopathy is an interesting premise –**

perhaps since the cohort of subjects used here seem to be amenable to multiple studies, the authors could consider looking at Tau PET study possibilities that can be corroborating this evidence and thereby providing evidence for their hypothesis. Moreover, as indicated earlier, there is a possibility that the authors did not observe any of the typical features of AD because of the early stage (~60 years) associated with the AD cohort – this needs to be considered in the discussion section if not pursued by the authors in their future studies to cement their hypothesis with evidence that may provide newer directions including looking at how GABAergic insults by the amyloidogenic proteins may need to be considered to get a complete understanding of the progression of the pathology.

We thank the reviewer for the insightful suggestion. Although as small fraction of participants in the current study did take part in tau imaging, we do not have enough power to test the hypothesis of contributory role of neuronal hyperexcitability as an early driver of tau pathology. However, this is one of our ongoing investigations and we will be sharing our findings in future manuscripts.

8. **This reviewer completely appreciates the limitation section that has addressed some of the above concerns, however, feels that there is a need to include the same at those different locations indicated so that there is continuity in the appreciation of the audience for the brevity of the study.**

Thank you for the suggestions. We have now addressed each of the points raised by the reviewer in prior items which greatly improved our manuscript from its original version.

9. **Lastly, this is a laudatory comment that allows the reviewer to appreciate the author's attention to detail where there were minimal, if any, errors associated with writing and representations in the manuscript allowing the latitude for the reviewer to provide a deeper insight into the scientific premise and perhaps constructive comments to enable the publication of this really good body of work.**

Thank you !

<u>Comments from the Scientific Editorial team:</u>

1. **Please supply a graphical abstract.**

We have included a graphical abstract following the specified criteria in this submission.

2. **Please add an Abbreviated Summary (up to 50 words) of your paper that captures the main purpose and conclusions of the work.**

We have included an abbreviated summary.

3. **Please include the appropriate checklist to comply with our ethical standards**

We have included the STROBE checklist with this submission.

4. **Data availability: Please provide the codes generated and used in this work within the supplementary material or through a repository, whose link has to be made available within the data availability statement.**

We have included the codes used for the analysis with specific instructions in the supplementary material.

5. **For figures 2-4: for each statistical test, please report in the figure legend, the N, test used, relevant test statistic, and exact p values (for example F or t values for ANOVAs and t-tests).**

We have now included the statistical details in the figure legends in Figures 3 and 5. Figure 2 and 4 do not represent statistical comparisons and only display average activity patterns.

6. **Table 3: Please be reminded that the tables should fit into one A4 page for the final manuscript version.**

We have revised table 3 accordingly.
