# Supplementary material for: Abnormal gamma phase-amplitude coupling in the parahippocampal cortex is associated with network hyperexcitability in Alzheimer’s disease
Source: Brain Commun. 2024 Apr 9;6(2):fcae121. doi: 10.1093/braincomms/fcae121 (PMC11043655; doi:10.1093/braincomms/fcae121)
Supplement: fcae121_Supplementary_Data [file Original Submission - Decision Letter.pdf › Original Submission - Decision Letter.pdf]

Original Submission - Decision Letter

30-Dec-2023

Dear Dr. Ranasinghe,

Thank you for your recent submission BRAINCOM-2023-534
entitled "Abnormal Gamma Phase-Amplitude Coupling in the Parahippocampal Cortex is Associated with Network Hyperexcitability in Alzheimer's Disease" to Brain Communications.

Your manuscript has been reviewed, and while the reviewers had largely positive comments, they have some constructive critiques that I think you could address in a minor revision of the manuscript. Therefore, I invite you to respond to the reviewer(s)' comments and revise your manuscript. The reviewer(s)' comments are included at the bottom of this message.

In addition to the reviewers' comments, please also address our Scientific Editor's comments added below to comply with our requirements for presentation of scientific data at Brain Communications.

Please format your revised manuscript according to our Instructions to Authors which can be found at: https://academic.oup.com/braincomms/pages/General_Instructions
A summary of key points is attached to help with this. Please note that there can be substantial delays to publication if you don't resubmit in line with our requirements.

To revise your manuscript, click the link below or log into https://mc.manuscriptcentral.com/braincom and enter your Author Centre, where you will find your manuscript title listed under "Manuscripts with Decisions." Under "Actions," click on "Create a Revision." Your manuscript number will be appended to denote a revision.

*** PLEASE NOTE: This is a two-step process. After clicking on the link, you will be directed to a webpage to confirm. ***

https://mc.manuscriptcentral.com/braincom?URL_MASK=4e0d19ceaa0547f88b86381d3c8821b1

Please upload a revised manuscript with the changes you have made shown either in coloured text or using "track changes".

When submitting your revised manuscript, you will be asked to respond to the reviewer(s) comments. Please be as specific as possible in your response to all of the comments.

The editorial team at Brain Communications also aims to increase transparency in scientific publishing and to demonstrate the value that peer review brings to the scientific process. Please therefore consider whether you are willing to have reviewer comments on your paper and your response published as supplementary data with the paper, if it is accepted. This is entirely optional and will only be done if both the authors and reviewers agree. When submitting your revision you will have the opportunity to update the answer you entered previously.

IMPORTANT: Your original files are available to you when you upload your revised manuscript. Please delete any redundant files before completing the submission.

Because we are trying to facilitate timely publication of manuscripts submitted to Brain Communications, we would appreciate your revised manuscript as soon as possible. If it is not possible for you to resubmit within 3 months, the option to submit a revision will expire, and we would then consider your revised paper as a new submission. However, if you need a little more time, please get in touch and we can extend this deadline for you.

Once again, thank you for submitting your manuscript to Brain Communications and I look forward to receiving your revision.

Best wishes

Dr. Rakez Kayed
Associate Editor, Brain Communications

Professor Tara Spires-Jones
Founding Editor, Brain Communications

Reviewer(s)' Comments to Author:

Reviewer: 1

Comments to the Author
This is a very interesting manuscript where authors use Phase-amplitude coupling (PAC) to access local gamma activity in the medial temporal lobe as a metric of neuronal excitability. Their results using a well designed human cohort are compelling and indicate specific patterns associated with network hyperexcitablity. This information can be very helpful to further examine hyperexcitability across different stages of pathology and its potential use as biomarker.
I have only minor comments:

In p11, Ln 20, it is not very clear whether the authors are referring to Primary age-related tauopathy. A more clear explanation in the text will help with clarity for the reader.

Please discuss why the left parahippocampal regions were more affected in AD

The manuscript appropriately use AD-EPI- and AD-EPI+ phenotypes to facilitate comparisons, but more likely than a binomial distribution, hyperexcitability is found as a continuum in the clinical settings where AD-EPI+ is at one end of this continuum. Indeed, proexcitatory synaptic imbalance in AD correlate with worse cognitive performance and more neuropathology across the continuum of AD (PMID: 36538112), please discuss the utility of PAC to explore this continuum and other brain regions.

Reviewer: 2 (Balaji Krishnan)

Comments to the Author
Phase Amplitude Coupling (PAC) and its concordance with magnetoencephaolography (MEG) and electroencephalography (EEG) associated characterization of a statistically well-powered group of AD subjects that can be divided into ones showing epileptic activity and with a control cohort has been attempted here to understand whether these parameters can be therapeutically translated to the clinic for diagnosing AD. Here are the major and minor points that may be important to consider for the current manuscript:

Major Points:
1) Page 6 - Line 1-8 - While the scientific premise of using PAC is innovative in this approach, it is not very commonplace for the journal readers to be able to appreciate the gravity of the approach as presented here. The authors may consider a sentence or two in the introduction that clearly highlights how PAC goes beyond earlier EEG studies both for future approaches as well as the possibility to mine previously obtained data.

2) Page 6 – Line 11-12 – The audience would benefit by the understanding of why the coupling of the specified frequencies are more beneficial than the conventionally used ones (if that is done). Moreover, it would also be very relevant for the authors to demonstrate how this range is ideal for the PAC analysis being considered here by either using previous references or by providing preliminary data that was done in earlier studies that refined the outcome thus allowing the authors to explore this premise.

3) Page 5 – Line 13-15 – This statement, in the opinion of this reviewer, seems to demonstrate a missed opportunity where the excitability alone was considered in the analysis. It is perhaps important for the authors to lay the rationale for why there was no focus on looking at signals that were statistically elevated in control subjects but missing in the AD patients which would have explained how the entorhinal PAC did not show any aberrant activity while being the foremost affected in AD patients.

4) Page 5 – Line 21-24 – The age group of subjects selected for the study is fairly early stages in the clinical progression of the disease – can the authors elaborate the practical or the scientific premise for why different age groups were not considered to allow the readers to appreciate the importance of the age range mentioned here.

5) Page 7 – Line 1-9 – The authors need to provide the distribution of male and female subjects in these studies at this level also in addition to the results section, since there is a strong disproportional representation of AD in females. This also raises the questions as to whether the authors need to consider sex/gender as a variable in interpreting the outcomes of the current study.

6) Page 7 – 11-25 – Can the authors elaborate on whether the studies on different patients were conducted at the same time of the day? There is a definitive component of circadian rhythm disruptions that needs to be considered for studying the progression of dementia. This necessitates that the authors, at the very least, consider reporting the time of the day and the state of the subjects (awareness/sleepiness) that the data was collected. Perhaps, if the statistical power remains intact, pooling the studies done at the same time of day with consideration to sex/gender may provide insights in PAC that may be more relevant from a clinical point of view.

7) Page 13 – Line 1-5 – The discussion by the authors to highlight the importance of neuronal excitability in the progression of AD-tauopathy is an interesting premise – perhaps since the cohort of subjects used here seem to be amenable to multiple studies, the authors could consider looking at Tau PET study possibilities that can be corroborating this evidence and thereby providing evidence for their hypothesis. Moreover, as indicated earlier, there is a possibility that the authors did not observe any of the typical features of AD because of the early stage (~60 years) associated with the AD cohort – this needs to be considered in the discussion section if not pursued by the authors in their future studies to cement their hypothesis with evidence that may provide newer directions including looking at how GABAergic insults by the amyloidogenic proteins may need to be considered to get a complete understanding of the progression of the pathology.

8) This reviewer completely appreciates the limitation section that has addressed some of the above concerns, however, feels that there is a need to include the same at those different locations indicated so that there is continuity in the appreciation of the audience for the brevity of the study.

9) Lastly, this is a laudatory comment that allows the reviewer to appreciate the author's attention to detail where there were minimal, if any, errors associated with writing and representations in the manuscript allowing the latitude for the reviewer to provide a deeper insight into the scientific premise and perhaps constructive comments to enable the publication of this really good body of work.

Comments from the Scientific Editorial team:

-Please supply a graphical abstract. A graphical abstract is a visual representation of the central finding or methodology of the paper. It will be displayed in the table of contents. Authors should strive to make the

graphical abstract informative, interesting, visually appealing, and straightforward (no caption needed, no abbreviations except gene names). The technical requirements for the graphical abstracts are:
- Size: 120 x 120 mm (300-600dpi) or 3000 x 3000 pixels
- Font:  Arial 12-16 pt
- File type: tiff or PDF
- Aspect ratio: 1:1 preferred

-Please add an Abbreviated Summary (up to 50 words) of your paper that captures the main purpose and conclusions of the work. The summary should state the main results written in the third person ('[1st author surname] et al. report that …') and conclusion. This summary will be used in the contents list online and should not contain any abbreviations other than those on the accepted abbreviations list and accepted gene/protein names, making it accessible to the non-specialist. Please upload it as a Word document. Allowed abbreviations: AIDS; ANOVA; AMPA; ATP; A,T,C,G; CNS; CSF; CT; DNA; ECG; EEG; EMG; GABA; HIV; MRI; NMDA; PET; PCR; RNA.

- Please include the appropriate checklist to comply with our ethical standards (see Reporting Guideline section within 'Instructions to authors' ).

-Data availability: Please provide the codes generated and used in this work within the supplementary material or through a repository, whose link has to be made available within the data availability statement.

-For figures 2-4: for each statistical test, please report in the figure legend, the N, test used, relevant test statistic, and exact p values (for example F or t values for ANOVAs and t-tests).

-Supp. Table 3: Please be reminded that the tables should fit into one A4 page for the final manuscript version.
